# The Nucleoporin CPR5 Modulates Plant Immunity via Guanylate‐Binding Proteins

**DOI:** 10.1111/mpp.70086

**Published:** 2025-04-27

**Authors:** Leiwen Pan, Shun Peng, Yuehui Zhang, Fenghui Xu, Xinyi Cai, Shen Liang, Qi Huang, Shilong Yu, Shui Wang

**Affiliations:** ^1^ Shanghai Engineering Research Center of Plant Germplasm Resources, Shanghai Collaborative Innovation Center of Plant Germplasm Resources, College of Life Sciences Shanghai Normal University Shanghai China; ^2^ National Engineering Laboratory of Crop Stress Resistance, College of Life Science Anhui Agricultural University Hefei Anhui China

**Keywords:** CPR5, guanylate‐binding protein, nuclear export signal, nuclear pore complex, plant immunity

## Abstract

The nuclear pore complex plays a key role in nuclear transport of immune signals. Two nucleoporins, CONSTITUTIVE EXPRESSION OF PR GENES 5 (CPR5) and GUANYLATE‐BINDING PROTEIN‐LIKE 3 (GBPL3), have been implicated in plant immunity. The *Arabidopsis* GBPL family comprises three members. While GBPL1 and GBPL3 were discovered to form an immune circuit, the role of GBPL2 remains unknown. Through genetic screening, we identified a gain‐of‐function mutation in *GBPL2*, named *suppressor of cpr5 23* (*scpr23*), that fully suppresses the *cpr5* mutant phenotype. The scpr23 function is attributed to a core amino acid residue within a nuclear export signal (NES) motif; its alteration causes a change of GBPL2 localisation from the endoplasmic reticulum to the nuclear envelope. Epistatic analysis demonstrated that knockdown of *GBPL3* expression in the *cpr5 scpr23* double mutant reverted its phenotype to that of the *cpr5* mutant, indicating that the function conferred by scpr23 is mediated by GBPL3. We further found that GBPL2 is a counterpart of mammalian ATLASTIN‐1, which belongs to a subfamily of the GBP proteins featuring transmembrane domains. Therefore, our findings reveal that the GBPL family proteins form a signalling complex that functions downstream of CPR5 to modulate plant immunity.

## Graphical abstract

The subcellular localisation of GBPL2 between the endoplasmic reticulum (ER) and nuclear envelope (NE) regulates the immune signalling pathway mediated by nucleoporins CPR5 and GBPL3 in *Arabidopsis*.

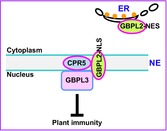

Plants have evolved two layers of innate immunity, pathogen‐associated molecular pattern (PAMP)‐triggered immunity (PTI) and effector‐triggered immunity (ETI), to defend themselves against pathogen invasion (Jones and Dangl 2006). CONSTITUTIVE EXPRESSION OF PR GENES 5 (CPR5) is a key negative regulator of plant ETI (Bowling et al. 1997; Wang et al. 2014; Zhang et al. 2025). CPR5 has been characterised as a membrane component of the nuclear pore complex (NPC). Usually, CPR5 proteins form oligomers to restrain the nuclear transport of immune signals and the core cell‐cycle regulators CYCLIN‐DEPENDENT KINASE INHIBITORs (CKIs). Upon pathogen infection, nucleotide‐binding leucine‐rich repeat receptors (NLRs) induce a conformational change in CPR5 from oligomer to monomer, promoting the nuclear influx of immune signals and the release of CKIs to activate immunity and programmed cell death (PCD) (Gu et al. 2016; Wang et al. 2014). CPR5 has further been characterised as an atypical RNA‐binding protein belonging to the serine and arginine‐rich (SR) family. The RNA splicing activator the NineTeen Complex (NTC) and the RNA polyadenylation factor CLEAVAGE AND POLYADENYLATION SPECIFICITY FACTOR (CPSF) form a ternary complex with CPR5 and function downstream of CPR5 to modulate plant immunity (Peng et al. 2022). These findings suggest that CPR5 serves as a nexus for integrating fundamental biological processes, such as nuclear transport, cell cycle progression, and RNA processing, to control plant ETI.

The guanylate‐binding protein (GBP) family, comprising crucial innate immune proteins induced by interferons, is extensively involved in the host's immune response against viruses and bacteria (Kim et al. 2016). The GBP‐like (GBPL) family in *Arabidopsis* consists of three members: GBPL1, GBPL2, and GBPL3. Like CPR5, GBPL3 has also been characterised as a component of the NPC (Tang et al. 2022). PWWP‐DOMAIN INTERACTOR OF POLYCOMBS1 (PWO1) is a key component of POLYCOMB REPRESSIVE COMPLEX 2 (PRC2), which is a chromatin‐associated methyltransferase catalysing methylation of histone H3 lysine 27 (H3K27me) (Hohenstatt et al. 2018). It has been shown that GBPL3 interacts with the nucleoskeleton proteins CROWDED NUCLEI (CRWNs) and PWO1, promoting H3K27me3‐dependent transcriptional repression (Reimann et al. 2023; Tang et al. 2022). Additionally, GBPL3 was shown to physically bind to the *SAR DEFICIENT1* (*SARD1*) and *CAM‐BINDING PROTEIN 60‐LIKE G* (*CBP60g*) genes, which are the master regulators of plant immunity (Huang et al. 2021; Kim et al. 2022). These findings highlight the role of GBPL3 in histone modification and the transcriptional regulation of *SARD1* and *CBP60g*. While GBPL1 and GBPL3 have been found to form an immune circuit, the role of GBPL2 remains unclear (Huang et al. 2021). In this study, we explored the role of GBPL proteins in the CPR5 signalling pathway.

The *Arabidopsis cpr5* mutant exhibits elevated salicylic acid (SA) content, auto‐immune activation, and spontaneous programmed cell death (PCD), resembling plant ETI (Bowling et al. 1997). To dissect the CPR5 signalling pathway, we conducted a genetic screen for *suppressors of cpr5* (*scpr*). Previously, we reported the top two candidates *scpr44* and *scpr57* (Peng et al. 2022). In this study, we further investigated the third candidate, *scpr23* (Figure 1a,b). The *SCPR23* gene was mapped to *GBPL2* (AT2G38840) through a combination of positional cloning and next‐generation sequencing (NGS) (Figure S1; Methods [Link], [Link]; Table [Link], [Link]). The phenotype of the *cpr5 scpr23* mutant was completely restored to that of the *cpr5* mutant by the *GBPL2* gene. Surprisingly, neither the T‐DNA insertion line (*gbpl2‐T*) nor the CRISPR/Cas9‐edited line (*gbpl2‐C*), both of which are null mutants with no GBPL2 protein expression, could suppress the *cpr5*‐induced phenotype, suggesting that *scpr23* is a semidominant gain‐of‐function mutation (Figure S1b–d). Further supporting this, the *scpr23* mutants exhibited severe sterility, which could be rescued by the *GBPL2* gene, whereas the *gbpl2* mutants remained fertile (Figure S1e).

**FIGURE 1:**
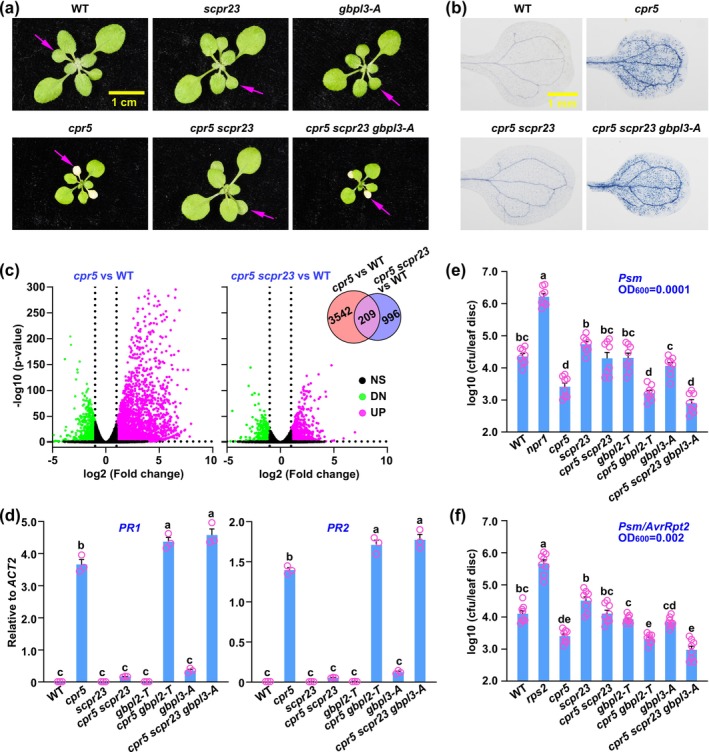
A gain‐of‐function mutation in the *GBPL2* gene suppresses the *cpr5* mutant through GBPL3 in *Arabidopsis*. (a) Two‐week‐old wild‐type (WT), *cpr5*, *scpr23*, *cpr5 scpr23*, *gbpl3‐A* and *cpr5 scpr23 gbpl3‐A* plants were photographed for early senescence (arrows). *gbpl3‐A*, an artificial microRNA‐based RNA‐silenced line of the *GBPL3* gene. (b) Cotyledons of 2‐week‐old plants were stained with trypan blue for cells that had undergone programmed cell death (PCD). (c) Two‐week‐old plants were used for RNA‐seq analysis. RNA‐seq data can be accessed in the SRA database under the Bioproject accession number PRJNA737003. Volcano plots illustrating differentially expressed genes (DEGs) of *cpr5* versus WT and *cpr5 scpr23* vs. WT. The *x*‐axis represents log_2_ FC (fold change) and the *y*‐axis represents −log_10_ *p*‐value. NS, not significant; UP, up‐regulated; DN, down‐regulated. Inset: Venn diagram shows the overlap between 3751 *cpr5*‐altered DEGs (FC > 2, *p* < 0.05) and 1205 *cpr5 scpr23*‐altered DEGs (FC > 2, *p* < 0.05). (d) Reverse transcription‐quantitative PCR was carried out on *PR1* (Left panel) and *PR2* (Right panel) in 12‐day‐old plants. *ACT2* was used as an internal control. Data are represented as mean ± SEM (*n* = 3). Statistical differences are indicated with letters (*p* < 0.01, one‐way analysis of variance (ANOVA) with Bonferroni post hoc test). (e) Four‐week‐old plants were inoculated with *Pseudomonas syringae* pv. *maculicola* ES4326 at an optical density (OD_600_) of 0.0001 (*Psm*, OD_600_ = 0.0001). Bacterial growth (colony‐forming unit, cfu) was measured 3 days later. Experiments were conducted three times with similar results. Data are represented as mean ± SEM (*n* = 8). Statistical differences are indicated with letters (*p* < 0.01, one‐way ANOVA with Bonferroni post hoc test). (f) Four‐week‐old plants were inoculated with *Psm*/*AvrRpt2* (OD_600_ = 0.002). Bacterial growth (cfu) was measured 3 days later. Experiments were conducted three times with similar results. Data are represented as mean ± SEM (*n* = 8). Statistical differences are indicated with letters (*p* < 0.01, one‐way ANOVA with Bonferroni post hoc test).

In mammals, guanylate‐binding proteins (GBPs), which are interferon‐induced GTPases, play a crucial role in cell‐autonomous innate immunity (Huang et al. 2019). *Arabidopsis* has three members in the GBP‐LIKE (GBPL) family. GBPL1 and GBPL3 have been implicated in plant immunity. It has been discovered that, similar to CPR5, GBPL3 is a nucleoporin, and the null *gbpl3* mutant is sterile (Huang et al. 2021; Tang et al. 2022). To explore the role of GBPL3 in the CPR5‐GBPL2 signalling pathway, we employed the artificial microRNA‐based RNA silencing method to generate *GBPL3* knockdown lines (referred to as *gbpl3‐A*) (Schwab et al. 2010). We identified several *gbpl3‐A* lines that exhibited a substantial decrease in transcription and protein levels compared to those in wild type plants (Figure S1f–i). Subsequently, we crossed *gbpl3‐A* with *cpr5 scpr23* to generate *cpr5 scpr23 gbpl3‐A* triple mutant. Surprisingly, we found that the *cpr5 scpr23* mutant was completely restored to the *cpr5* mutant phenotype by *gbpl3‐A* (Figure 1a,b). These data suggest that GBPL3 acts downstream of *scpr23* to inhibit the *cpr5*‐induced PCD.

RNA‐seq analysis revealed that the majority of the CPR5‐regulated differentially expressed genes (DEGs) (3542/3751; 94.43%) are dependent on *scpr23* (Figure 1c). Gene ontology (GO) enrichment analysis found that plant defence is the top signalling pathway (*p* = 3.9e−94) among the CPR5‐regulated and scpr23‐dependent DEGs (Figure S1j,k). Reverse transcription‐quantitative PCR (RT‐qPCR) of plant immune response marker genes *PR1* and *PR2* validated that the *cpr5*‐induced immune response was suppressed by *scpr23*, which was mediated by GBPL3 (Figure 1d). To assess the role of the *GBPL* genes in plant immunity, we conducted pathogen infections using the virulent pathogen *Pseudomonas syringae* pv. *maculicola* ES4326 (Psm) and the avirulent pathogen Psm carrying the effector gene *AvrRpt2* (Psm*/AvrRpt2*). The results indicated that both the *cpr5*‐induced basal immunity and ETI were suppressed in the *cpr5 scpr23* mutant but restored in the *cpr5 scpr23 gbpl3‐A* mutant (Figure 1e,f). These data suggest that GBPL3 acts downstream of *scpr23* to inhibit *cpr5*‐induced immune responses.

Transient expression assays in *Nicotiana benthamiana* revealed that wild‐type GBPL2 localised predominantly to the endoplasmic reticulum (ER), whereas the GBPL2^L586F^ (scpr23) mutant protein exhibited enriched localisation at the nuclear envelope (Figure 2a,b). A similar distribution pattern was observed for GBPL2 and scpr23 in *Arabidopsis*. Notably, nuclear VENUS fluorescence signals were detected in *scpr23‐VENUS*‐transgenic *Arabidopsis* plants but were absent in *GBPL2‐VENUS*‐transgenic plants (Figure 2c). CPR5 colocalised with GBPL2 in the nucleus (Figure S2a). Bimolecular fluorescence complementation (BiFC) assays demonstrated reciprocal interactions among CPR5, GBPL2, and GBPL3 (Figure 2d,e). The leucine residue at position 586 (L586), substituted with phenylalanine (F) in the *scpr23* mutant (L586F), is highly conserved in GBPL2 across plant species and forms part of a predicted leucine‐rich nuclear export signal (NES). This NES motif lies adjacent to a nuclear localisation signal (NLS) (Figure 2f). To confirm the functional importance of the NES in GBPL2 localisation and activity within the *cpr5* mutant background, we generated two constructs: *GBPL2‐NES‐M* (with mutations in the NES core residues) and *scpr23‐NES* (fused to a canonical NES from human PKIα) (Wen et al. 1995). Consistent with NES disruption, *GBPL2‐NES‐M* phenocopied *scpr23*, suppressing the *cpr5* mutant phenotype. Conversely, *scpr23‐NES* restored wild‐type function, complementing the *scpr23* mutant (Figure 2g; Figure S2b). These findings indicate that the *scpr23* mutation disrupts the NES motif in *GBPL2*, causing ER‐to‐nuclear envelope mislocalisation and conferring a gain‐of‐function ability to activate GBPL3, thereby suppressing the *cpr5* mutant phenotype.

**FIGURE 2:**
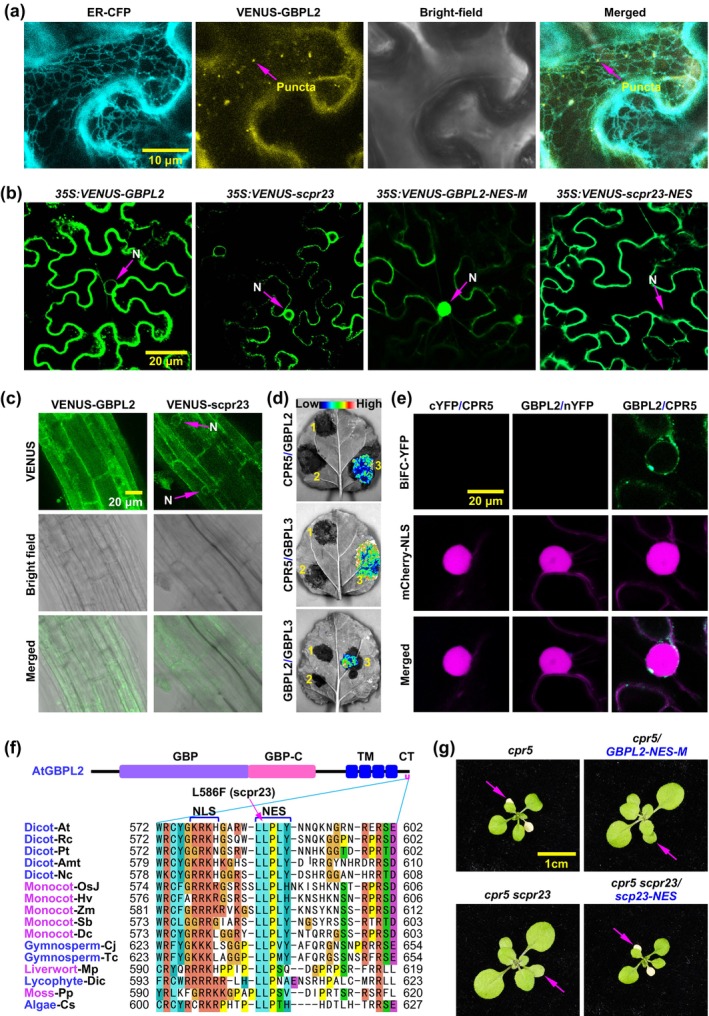
The phenotype conferred by the *scpr23* mutant is attributed to the disruption of a nuclear export signal in the C‐terminus of the GBPL2 protein. (a) Subcellular localisation of GBPL2 protein in *Nicotiana benthamiana*. Agrobacteria carrying the constructs of *ER‐CFP* (an endoplasmic reticulum [ER] marker, WAK2‐SP‐CFP‐HDEL, the signal peptide of *Arabidopsis thaliana* wall‐associated kinase 2 and the ER retention signal His‐Asp‐Glu‐Leu are at the N‐terminus and the C‐terminus of cyan fluorescent protein, respectively) (Nelson et al. 2007) and *35S:GBPL2‐VENUS* were co‐infiltrated into *N. benthamiana* leaves. Puncta on the ER is indicated (arrow). (b) Subcellular localisation of VENUS‐tagged proteins in *N. benthamiana*. Agrobacteria carrying constructs of *35S:VENUS‐GBPL2*, *35S:VENUS‐scpr23*, *35S:VENUS‐GBPL2‐NES‐M* (where the putative core nuclear export signal [NES] motif “LLPLY” is substituted by “LDADY”), and *35S:VENUS‐scpr23‐NES* (where the NES motif “LALKLAGLDI” from human PKIα protein is fused to the C‐terminus of the scpr23 protein) were infiltrated into *N. benthamiana*. Observation was conducted after 2 days. Nucleus (N) is indicated (arrow). (c) Subcellular localization of VENUS‐tagged proteins in *Arabidopsis*. Roots of transgenic *Arabidopsis* plants expressing *NP:VENUS‐GBPL2* (*NP*, native promoter) and *NP:VENUS‐scpr23* were imaged to determine protein localisation. Nucleus (N) is indicated (arrow). (d) The bimolecular fluorescence complementation‐luciferase (BiFC‐LUC) assay was performed by transiently co‐expressing split‐LUC fusion proteins in *N. benthamiana* leaves for 2 days to detect interactions between proteins. Luciferase was split into N‐terminal LUC (nLUC) and C‐terminal LUC (cLUC). Empty vectors of nLUC (designated by the number “1”) and cLUC (“2”) were used as negative controls. Each pair of proteins was tested with nLUC fused to one protein's C‐terminus and cLUC fused to the other protein's N‐terminus (“3”). The colour bar indicates signal intensity. (e) In the BiFC‐YFP assay, we transiently co‐expressed *35S:mCherry‐NLS* (mCherry‐NLS), *35S:CYFP‐CPR5* (CPR5, where the C‐terminus of YFP was fused to the N‐terminus of the CPR5 protein), and *35S:GBPL2‐nYFP* (GBPL2, where the N‐terminus of YFP is fused to the C‐terminus of the GBPL2 protein) in *N. benthamiana* for 2 days. The mCherry‐NLS (where the nuclear localisation signal [NLS] of SV40 protein was fused to the C‐terminus of mCherry) served as a nuclear marker. The empty vectors *35S:nYFP* (*nYFP*) and *35S:cYFP* (*cYFP*) were used as negative controls. (f) Top panel: The GBPL2 protein structure. CT, C‐terminus; GBP, guanylate‐binding protein; GBP‐C, GBP‐C‐terminal helical domain; TM, transmembrane domain. Bottom panel: Alignment of the C‐termini of GBPL2 proteins: Includes dicots *Amborella trichopoda* (*Amt*), *Arabidopsis thaliana* (*At*), *Nymphaea colorata* (*Nc*), *Populus trichocarpa (Pt)*, and *Ricinus communis* (*Rc*); monocots *Dendrobium catenatum* (*Dc*), *Hordeum vulgare* (*Hv*), *Oryza sativa Japonica* (*OsJ*), *Sorghum bicolor* (*Sb*), *and Zea mays* (*Zm*); gymnosperms *Cryptomeria japonica* (*Cj*), and *Taxus chinensis* (*Tc*); club‐mosses *Diphasiastrum complanatum* (*Dic*); mosses *Physcomitrium patens* (*Pp*); liverworts *Marchantia polymorpha* (*Mp*); green algae *Closterium sp* (*Cs*). The putative NLS, NES, and *scpr23* mutation (L586F) are indicated. (g) Two‐week‐old *cpr5*, *cpr5*/*GBPL2‐NES‐M*, *cpr5 scpr23*, *cpr5 scpr23*/*scpr23‐NES* plants were photographed for early senescence (arrows). *GBPL2*, the *GBPL2* gene; *GBPL2‐NES‐M*, the NES‐mutated *GBPL2* gene; *scpr23‐NES*, the *scpr23* plus an NES from the PKIα protein.

The expression levels of *PR1* and *PR2* defence genes showed no significant change in *gbpl2* mutants relative to wild‐type plants but were markedly elevated in *cpr5 gbpl2* double mutants compared to *cpr5* single mutants (Figure 1d). Overexpression of *GBPL2* had no observable effect on the morphological phenotype of either wild‐type or *cpr5* plants (Figure S2c). While *PR1* and *PR2* transcript levels remained unaltered in the wild‐type background, they were substantially higher in *cpr5* mutants overexpressing *GBPL2* (Figure S2d). These findings indicate that CPR5 and GBPL2 act cooperatively to regulate plant immunity, as the immunomodulatory role of GBPL2 becomes evident only in the *cpr5* mutant background.

To further explore the underlying mechanism by which *scpr23* suppresses the *cpr5*‐induced phenotype, we conducted a genetic screen for the *recurrence of cpr5* (*rcpr*) (Figure 3a). The identified 11 *rcpr* candidates all displayed mutations in the *GBPL2* gene. Among them, eight lines were either due to the disruption of the intron splicing sites or the generation of premature stop codons, while three lines were caused by the alteration of the encoded amino acid residues (G132E, C411Y and P454L). All these three amino acid residues are highly conserved in GBPL2 proteins throughout the plant kingdom, indicating their significance for GBPL2 function. In *rcpr5*, we identified a substitution where glycine was replaced by glutamic acid at position 132 (G132E), transforming the P‐loop motif from GxxxxGKS to GxxxxEKS. There are two P‐loop motifs (P‐loop1 and P‐loop2) in the GBPL2 protein, and the altered one is adjacent to the G3 box (DxxG), which is a distinctive feature of plant GBPL2 proteins. This alteration implies a disruption in the GTP‐binding activity (Wittinghofer and Vetter 2011). In *rcpr6*, we noticed a mutation from tryptophan (W) to a stop codon at 584 (W584*), which is very close to the L586F alteration in the *scpr23* mutant (Figure 3b,c; Figure S3). Based on the three‐dimensional protein structure predicted by AlphaFold, the P‐loop1 motif is spatially close to the P‐loop2 motif, and so is C411 to P454, suggesting that each pair of them might constitute a functional domain within the GBPL2 protein (Figure 3d). These findings indicate that GBPL2 functions as a GTPase and the function conferred by scpr23 represents a gain of function rather than a disruption of its C‐terminal domain.

**FIGURE 3:**
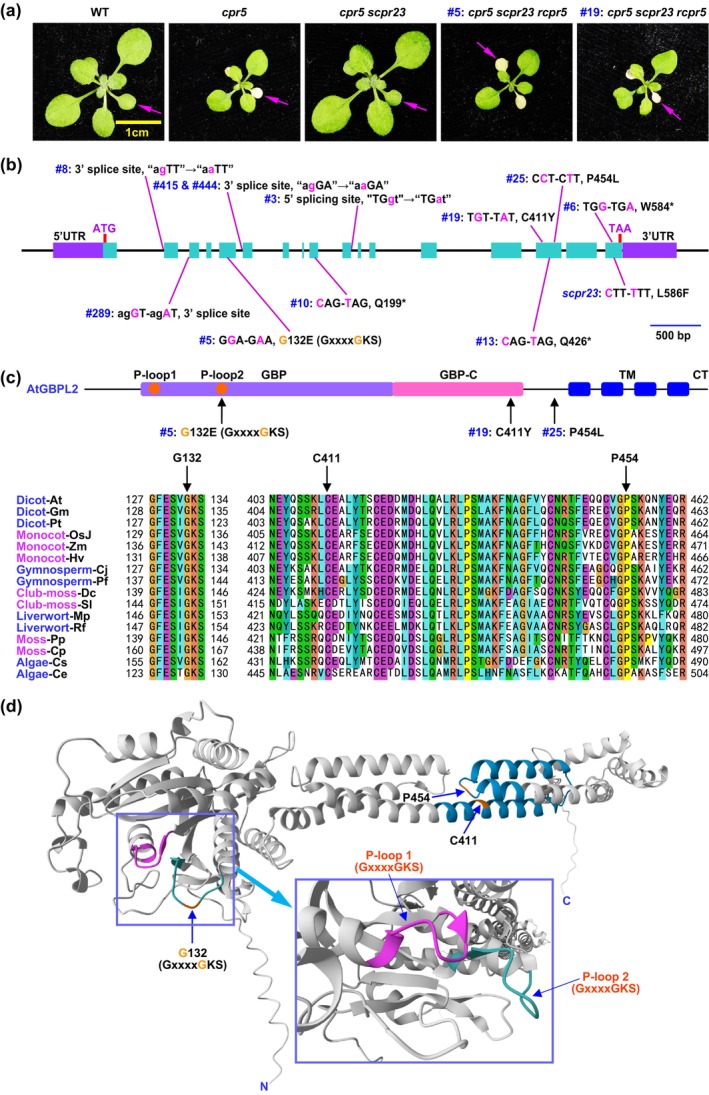
The GTPase activity is essential for the *scpr23* suppression of *cpr5* mutant. (a) Two‐week‐old wild‐type (WT), *cpr5*, *cpr5 scpr23*, and two lines of *cpr5 scpr23 rcpr* (*recurrence of cpr5*: #5 and #19) plants were photographed for early senescence (arrows). (b) Detailed information on the 11 sequenced *recurrence of cpr5* (*rcpr*) lines. (c) Top panel: The GBPL2 protein structure. There are two P‐loop motifs (P‐loop1 and P‐loop2: GxxxxGKS) present in the GBPL2 protein. CT, C‐terminus; GBP, guanylate‐binding protein; GBP‐C, GBP‐C‐terminal helical domain; TM, transmembrane domain. The positions of three *rcpr* lines, namely #5, #19 and #25, are indicated. Bottom panel: Alignment of the regions of GBPL2 proteins around the *rcpr* lines #5, #19 and #25: Includes dicots such as *Arabidopsis thaliana* (*At*), *Glycine max* (*Gm*), and *Populus trichocarpa (Pt)*; monocots such as *Hordeum vulgare* (*Hv*), *Oryza sativa Japonica* (*OsJ*), *and Zea mays* (*Zm*); gymnosperms *Cryptomeria japonica* (*Cj*) and *Pinus flexilis* (*Pf*); club‐mosses such as *Diphasiastrum complanatum* (*Dic*) and *Selaginella lepidophylla* (*Sl*); mosses such as *Physcomitrium patens* (*Pp*) and *Ceratodon purpureus* (*Cp*); liverworts such as *Marchantia polymorpha* (*Mp*) and *Riccia fluitan* (*Rf*); algae such as *Closterium sp* (*Cs*) and *Chlamydomonas eustigma* (*Ce*). The positions of the amino acid residues G132, C411 and P454 (where are mutated in the *rcpr* lines #5, #19 and #25, respectively) are indicated. (d) The three‐dimensional structure of *Arabidopsis* GBPL2 protein was predicted by AlphaFold at https://www.alphafold.ebi.ac.uk/. The regions of GBPL2 proteins around the *rcpr* lines #19 and #25 showed in Figure 3c are coloured light blue. The positions of amino acid residues G132, C411 and P454 are indicated. Inset: A close‐up showing the two P‐loop motifs (P‐loop1 and P‐loop2: GxxxxGKS).

Some of the GBP family proteins contain transmembrane (TM) domains in the C‐terminus, such as *Arabidopsis* GBPL2 and the ROOT HAIR DEFECTIVE3 (RHD3) subfamily, yeast SYNTHETIC ENHANCEMENT OF YOP1 (SEY1), and human Atlastin (ATL) subfamily (Figure 4a) (Huang et al. 2019). Based on the fingerprint sequences of GTP‐binding motifs (G1‐G5), these GBP proteins are categorised into two groups: the human Atlastin (ATL) group and the yeast SEY1 group. GBPL2 shows more similarity to human ATL proteins, while RHD3 is more akin to yeast SEY1 (Figure 4b,c; Figures S3 and S4) (Wittinghofer and Vetter 2011). Yeast SEY1 was demonstrated to be functionally replaceable by *Arabidopsis* RHD3 (Zhang et al. 2013). ATL1 and GBPL2 proteins share a putative NES domain adjacent to an NLS in their C‐termini (Figure 4d). Introduction of *SEY1* and *ATL1* into *cpr5 scpr23* mutants did not rescue the *scpr23* mutation. In contrast, replacing the putative NES motif of ATL1 with that of GBPL2 led to partial complementation of the *scpr23* mutant (Figure 4e). These data suggest that GBPL2 acts as the plant counterpart of animal ATL1 and highlight the importance and the distinctive feature of the NES motif for GBPL2 and ATL1.

**FIGURE 4:**
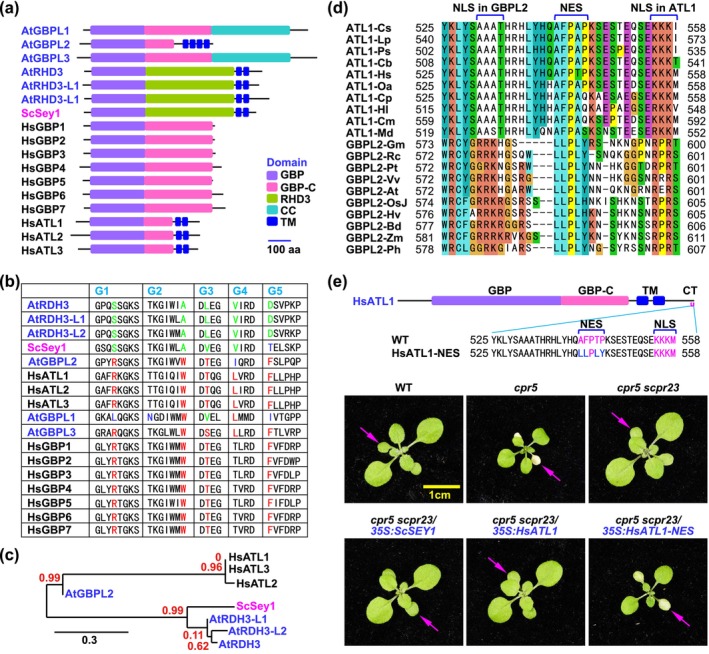
Plant GBPL2 functions as an orthologue of animal ATL1. (a) Schematic diagram illustrates the domain structures of the GBP family proteins, including GBPL subfamily (*Arabidopsis*), RDH3 subfamily (*Arabidopsis*), SEY1 (yeast), GBP subfamily (human), and ATL subfamily (human) proteins. *At*, *Arabidopsis thaliana* ; *Hs*, *Homo sapiens* ; *Sc*, *Saccharomyces cerevisiae* ; GBP, guanylate‐binding protein; GBP‐C, GBP‐C‐terminal helical domain; CC, coiled‐coil domain; RHD3, ROOT HAIR DEFECTIVE 3 domain; TM, transmembrane domain. (b) The fingerprint sequences of G1–G5 motifs in the GTPase domains of the GBP family proteins. (c) The phylogenetic tree of the GBP family proteins was constructed at http://www.phylogeny.fr/ based on the fingerprint sequences of the G1–G5 motifs in (b). (d) Alignment of the C‐termini of animal ATL1 and plant GBPL2 proteins, with the putative nuclear export signal (NES) indicated. NLS, nuclear localisation signal. Animal species include *Camelus bactrianus* (*Cb*), *Chelonia mydas* (*Cm*), *Crocodylus porosus* (*Cp*), *Ceratotherium simum* (*Cs*), *Haliaeetus leucocephalus* (*Hl*), *Homo sapiens* (*Hs*), *Lynx pardinus* (*Hp*), *Monodelphis domestica* (*Md*), *Ornithorhynchus anatinus* (*Oa*), and *Phocoena sinus* (*Ps*); plant species include *Arabidopsis thaliana* (*At*), *Brachypodium distachyon* (*Bd*), *Glycine max* (*Gm*), *Hordeum vulgare* (*Hv*), *Oryza sativa japonica* (*OsJ*), *Panicum hallii* (*Ph*), *Populus trichocarpa* (*Pt*), *Ricinus communis* (*Rc*), *Vitis vinifera* (*Vv*), *Zea mays* (*Zm*). (e) Top panel: Schematic diagram illustrating the structure of the human ATL1 (HsATL1) protein. The putative core NES motif AFPTP in HsATL1 is substituted by LLPLY in HsATL1‐NES. WT, wild type. Bottom panel: Two‐week‐old plants were photographed for early senescence (arrows).

Our genetic and cell biology data (Figures 1 and 2; Figures S1 and S2) suggest that the functional output of GBPL2 downstream of CPR5, specifically its role in the *cpr5* phenotype, depends on the ratio of GBPL2 protein distribution between the ER and the nuclear envelope (ER/NE). In *cpr5 gbpl2* mutants, the absence of GBPL2 has no effect on the *cpr5* phenotype (Figure S1c). By contrast, in *cpr5 scpr23* mutants, the scpr23 (GBPL2^L586F^) accumulates predominantly in the NE, reducing the ER/NE ratio and thereby suppressing the *cpr5* phenotype (Figures 1a and 2b). Conversely, reintroducing wild‐type GBPL2 into the *cpr5 scpr23* mutant increases the ER/NE ratio (due to GBPL2's ER localisation), which restores the *cpr5* phenotype (Figure 2b; Figure S1c). This model is further supported by experiments manipulating GBPL2 localisation. Disrupting the NES motif in GBPL2 (LLPLY to LDADY) forced NE localisation of the mutant protein and suppressed the *cpr5* phenotype. Conversely, fusing the human PKIα NES motif (LALKLAGLDI) to the C‐terminus of scpr23 restored ER localisation of the chimeric protein and reinstated the *cpr5* phenotype in *cpr5 scpr23* mutants (Figure 2g; Figure S2b). These findings highlight the importance of dynamic compartmentalisation in regulating protein function, paralleling mechanisms observed in plant immunity. For example, the pathogen effector AvrRps4 modulates the subcellular localisation of the EDS1–RPS2 complex. Nuclear localisation of this complex activates immune signalling, while cytoplasmic accumulation triggers cell death (Heidrich et al. 2011).

Both CPR5 and GBPL3 have been identified as components of the NPC and play a crucial role in plant immunity (Gu et al. 2016; Huang et al. 2021; Kim et al. 2022; Peng et al. 2022; Reimann et al. 2023; Tang et al. 2022; Wang et al. 2014). In this study, we uncovered a genetic link between CPR5 and GBPL3 through *scpr23*, a gain‐of‐function mutation in *GBPL2*, suggesting that GBPL2 is involved in the CPR5–GBPL3 signalling pathway (Figure 1). Our data demonstrated that GBPL2 functions as a GTPase, probably an orthologue of the animal ATL1 protein. Both plant GBPL2 and animal ATL1 proteins belong to a subfamily of the GBP proteins featuring transmembrane domains (Figures 2, 3, 4; Figures S3 and S4). ATL proteins facilitate homotypic fusion of ER membranes in metazoans (Yan et al. 2015). Similarly, GBPL2 proteins are also localised to the ER (Figure 2a). A disruption in a putative NES motif of the GBPL2 protein allows it to relocate to the nuclear envelope, resulting in the suppression of *cpr5*‐induced immunity and plant sterility (Figures 1 and 2; Figure S1). This discovery highlights the important role of GBPL2 translocation between the nuclear envelope and the ER in both immunity and development. While the subcellular localisation of the scpr23 protein (GBPL2^L586F^) is involved in the CPR5–GBPL3 signalling, the function of the wild‐type GBPL2 has yet to be explored.

## Conflicts of Interest

The authors declare no conflicts of interest.

## Supporting information


**Figure S1.** Cloning and characterisation of the *SCPR23* gene.

**Figure S2.** Overexpression of *GBPL2* in wild‐type and *cpr5* plants.

**Figure S3.** Alignment of plant GBPL2, plant RHD3 and animal ATL1 proteins.

**Figure S4.** The phylogenetic tree of plant GBPL2, plant RHD3 and animal ATL1 proteins.

**Methods S1.** Experimental procedures.

**Table S1.** The exonic and nonsynonymous single‐nucleotide polymorphisms (SNPs) around the *SCPR23* gene identified through the next‐generation sequencing (NGS) analysis.

## Data Availability

The data that support the findings of this study are available from the corresponding author upon reasonable request.
