# Supplementary material for: The Nucleoporin CPR5 Modulates Plant Immunity via Guanylate‐Binding Proteins
Source: Mol Plant Pathol. 2025 Apr 27;26(4):e70086. doi: 10.1111/mpp.70086 (PMC12034427; doi:10.1111/mpp.70086)
Supplement: Supplementary file 1 — Figure S1. Cloning and characterisation of the SCPR23 gene. [file MPP-26-e70086-s004.pdf]

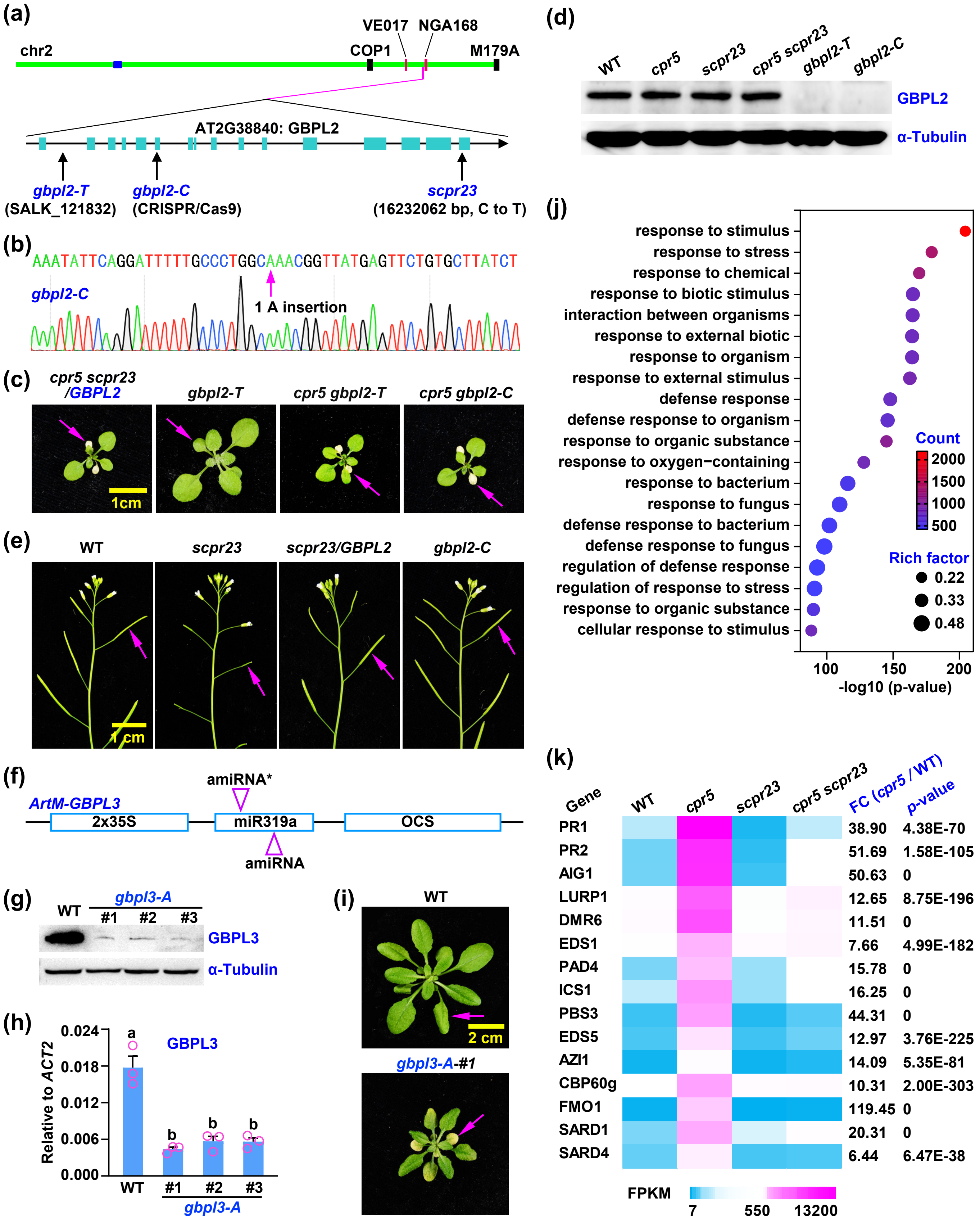
(a)
VE017
NGA168
chr2
COP1
M179A
AT2G38840: GBPL2
gbpl2-T
(SALK_121832)
gbpl2-C
(CRISPR/Cas9)
scpr23
(16232062 bp, C to T)
(b)
AAATATTCAGGATTTTTGCCCTGGCAAACGGTTATGAGTTCTGTGCTTATCT
gbpl2-C
1 A insertion
(c)
cpr5 scpr23 /GBPL2
gbpl2-T
cpr5 gbpl2-T
cpr5 gbpl2-C
1cm
(d)
WT
cpr5
scpr23
cpr5 scpr23
gbpl2-T
gbpl2-C
GBPL2
α-Tubulin
(e)
WT
scpr23
scpr23/GBPL2
gbpl2-C
1 cm
(f)
ArtM-GBPL3
2x35S
amiRNA*
miR319a
amiRNA
OCS
(g)
WT
gbpl3-A
#1
#2
#3
GBPL3
α-Tubulin
(h)
Relative to ACT2
0.024
0.018
0.012
0.006
0.000
GBPL3
a
b
b
b
WT
#1
#2
#3
gbpl3-A
(i)
WT
2 cm
gbpl3-A-#1
(j)
response to stimulus
response to stress
response to chemical
response to biotic stimulus
interaction between organisms
response to external biotic
response to organism
response to external stimulus
defense response
defense response to organism
response to organic substance
response to oxygen−containing
response to bacterium
response to fungus
defense response to bacterium
defense response to fungus
regulation of defense response
regulation of response to stress
response to organic substance
cellular response to stimulus
Count
2000
1500
1000
500
Rich factor
0.22
0.33
0.48
100
150
200
-log10 (p-value)
(k)
Gene
WT
cpr5
scpr23
cpr5 scpr23
FC (cpr5 / WT)
p-value
PR1 38.90 4.38E-70
PR2 51.69 1.58E-105
AIG1 50.63 0
LURP1 12.65 8.75E-196
DMR6 11.51 0
EDS1 7.66 4.99E-182
PAD4 15.78 0
ICS1 16.25 0
PBS3 44.31 0
EDS5 12.97 3.76E-225
AZI1 14.09 5.35E-81
CBP60g 10.31 2.00E-303
FMO1 119.45 0
SARD1 20.31 0
SARD4 6.44 6.47E-38
FPKM
7
550
13200

FIGURE S1. Cloning and characterization of the *SCPR23* gene.

(a) Positional cloning located the *SCPR23* gene within an approximately 846-kb region on chromosome 2, between the cleaved amplified polymorphic sequence (CAPS) marker *VE017* (chr2, 15445461 bp) and the simple sequence length polymorphism (SSLP) marker *NGA168* (chr2, 16291841 bp). Next-generation sequencing (NGS) analysis revealed four single nucleotide polymorphisms (SNPs) in this region. One of these SNPs (chr2, 16232062 bp) resulted in a C to T substitution in the final exon, causing a nonsense mutation (changing the genetic code from CTT to TTT) and leading to an amino acid change from L to F at position 586. This mutation impacts the gene encoding GUANYLATE-BINDING PROTEIN-LIKE 2 (GBPL2). A T-DNA insertion line (SALK_121832), designated as *gbpl2-T*, is located in the first intron of the *GBPL2* gene, while a CRISPR/Cas9-edited line, designated as *gbpl2-C*, is positioned in the sixth exon of this gene.

(b) The CRISPR/Cas9-edited lines of the *GBPL2* gene (*gbpl2-C*).

(c) Two-week-old *cpr5 scpr23*/*GBPL2*, *gbpl2-T*, *cpr5 gbpl2-T*, and *cpr5 gbpl2-C* plants were photographed for early senescence (arrows). *GBPL2*, the *GBPL2* gene; *gbpl2-C*, a CRISPR/Cas9-edited line.

(d) Total proteins of two-week-old WT, *cpr5*, *scpr23*, *cpr5 scpr23*, *gbpl2-T*, and *gbpl2-C* plants were blotted with anti-GBPL2 and anti-α-tubulin. The α-tubulin protein served as a loading control.

(e) The inflorescences of WT, *scpr23*, *scpr23/GBPL2* and *gbpl2-C* were photographed. Siliques are indicated by arrows.

(f) Schematic diagram depicts the structure of *ArtM-GBPL3*, an artificial microRNA construct. The amiRNA and amiRNA* sequences for targeting the *GBPL3* gene were predicted at http://wmd3.weigelworld.org. The primers used for the construction of *ArtM-GBPL3* are listed in S3 Table.

(g) Total proteins of 12-day-old WT and *gbpl3-A* (three lines: #1-#3, as described in Additional file 1: Fig. S2B) plants were blotted with anti-α-Tubulin and anti-GBPL3. The anti-GBPL3 antibody was raised against a synthesized C-terminal fragment of

GBPL3 protein (comprising amino acids 1069-1082: REEERKKQREVTSS) as previously described (Huang et al., 2021). It was produced by Qiwei Yicheng Technology (Beijing, China).

(h) RT-qPCR was carried out on *GBPL3* in 12-day-old WT and *gbpl3-A* (three lines: #1-#3) plants. *ACT2* was used as an internal control. Data are represented as mean ± SEM (n = 3). Statistical differences are indicated with letters ($P < 0.01$, one-way ANOVA with Bonferroni post hoc test).

(i) Twenty-four-day-old WT and *gbpl3-A* plants were photographed for early senescence (arrows).

(j) GO enrichment analysis of 3,542 DEGs which are altered in *cpr5* mutants (*cpr5* versus WT, $P < 0.05$, FC > 2) and depend on *scpr23* (*cpr5 scpr23* versus WT, FC < 2, $P > 0.05$). The bubble chart shows biological process enrichment of DEGs. The y-axis represents biological process. The x-axis represents the enrichment significance (–log10 P-value). Size of the bubble represents rich factor, which is the ratio of the amount of DEGs enriched in a biological process and the amount of all genes annotated in this biological process.

(k) Heatmap showing RNA-seq data of plant immune marker genes, including the defense markers such as *PATHOGENESIS-RELATED 1* (*PR1*), *PR2*, *AVRRPT2-INDUCED GENE 1* (*AIG1*), *LATE UPREGULATED IN RESPONSE TO HYALOPERONOSPORA PARASITICA 1* (*LURP1*), *DOWNY MILDEW RESISTANT 6* (*DMR6*), the core immune regulators such as *ENHANCED DISEASE SUSCEPTIBILITY 1* (*EDS1*) and *PHYTOALEXIN DEFICIENT 4* (*PAD4*), the key SA biosynthesis enzymes and regulators such as *ISOCHORISMATE SYNTHASE 1* (*ICS1*), *AVRPPHB SUSCEPTIBLE 3* (*PBS3*), and EDS5, as well as the key systemic acquired resistance (SAR) regulators such as *AZELAIC ACID INDUCED 1* (*AZI1*), *CALMODULIN BINDING PROTEIN 60-LIKE g* (*CBP60g*), *FLAVIN-DEPENDENT MONOOXYGENASE 1* (*FMO1*), *SAR DEFICIENT 1* (*SARD1*), and *SARD4*. The FC of cpr5/WT as well as its P-values are indicated.
