# Supplementary material for: The Nucleoporin CPR5 Modulates Plant Immunity via Guanylate‐Binding Proteins
Source: Mol Plant Pathol. 2025 Apr 27;26(4):e70086. doi: 10.1111/mpp.70086 (PMC12034427; doi:10.1111/mpp.70086)
Supplement: Supplementary file 2 — Figure S2. Overexpression of GBPL2 in wild‐type and cpr5 plants. [file MPP-26-e70086-s001.pdf]

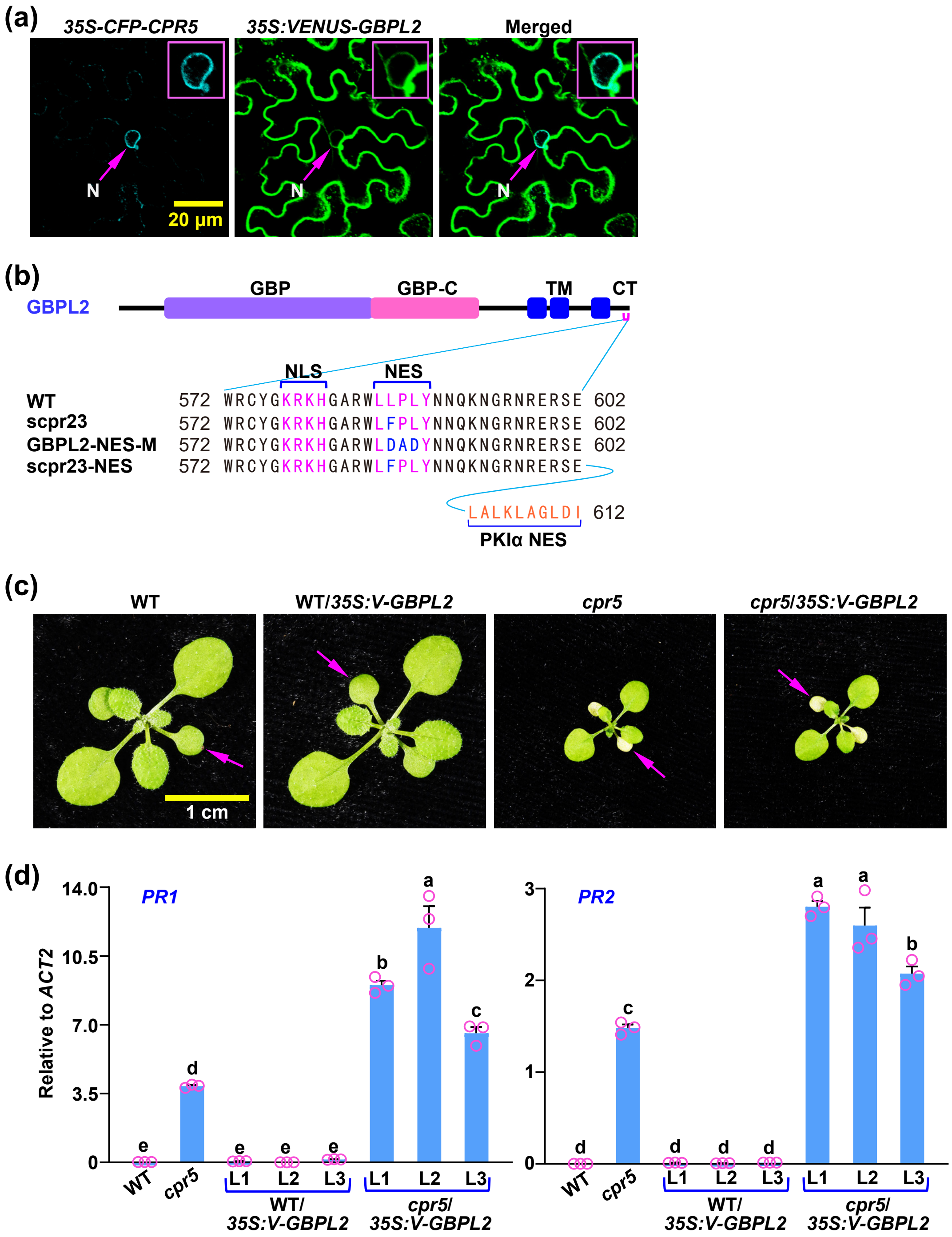

(a)
35S-CFP-CPR5
35S:VENUS-GBPL2
Merged
N
20 µm
(b)
GBPL2
GBP
GBP-C
TM
CT
NLS
NES
WT 572 WRCYGKRKHGARWLLPLYNNQKNGRNRERSE 602
scpr23 572 WRCYGKRKHGARWLFPLYNNQKNGRNRERSE 602
GBPL2-NES-M 572 WRCYGKRKHGARWLDADYNNQKNGRNRERSE 602
scpr23-NES 572 WRCYGKRKHGARWLFPLYNNQKNGRNRERSE
LALKLAGLDI 612
PKIα NES
(c)
WT
WT/35S:V-GBPL2
cpr5
cpr5/35S:V-GBPL2
1 cm
(d)
PR1
PR2
Relative to ACT2
14.0
10.5
7.0
3.5
0
3
2
1
WT
cpr5
L1
L2
L3
WT/
35S:V-GBPL2
cpr5/
35S:V-GBPL2

FIGURE S2. Colocalization of CPR5 and GBPL2, and *GBPL2* overexpression in wild-type and *cpr5* plants.

(a) Colocalization analysis was performed by transiently co-expressing *35S:CFP-CPR5* (with CFP fused to the N-terminus of CPR5) and *35S:VENUS-GBPL2* (with VENUS fused to the N-terminus of GBPL2; see in Fig. 2b) in *N. benthamiana* for two days. Inset (pink box): An enlarged view of the nucleus (N, arrow) is shown.

(b) The C-terminus sequences of GBPL2 (WT), scpr23, GBPL2-NES-M (the putative core NES motif "LLPLY" is substituted by "LDADY"), and scpr23-NES (the NES motif, LALKLAGLDI, from human PKIα protein fused to the C-terminus of the scpr23 protein) proteins. The putative NLS and NES motifs are indicated.

(c) Two-week-old wild-type (WT), WT/*35S:V-GBPL2* (expressing the *35S* promoter-driven *VENUS-GBPL2* fusion gene in a WT background), *cpr5*, and *cpr5*/*35S:V-GBPL2* plants were photographed to assess early senescence (arrows).

(d) RT-qPCR analysis of *PR1* (left panel) and *PR2* (right panel) expression in 12-day-old WT, *cpr5*, WT/*35S:V-GBPL2* (three independent transgenic lines, L1–L3), and *cpr5*/*35S:V-GBPL2* (three lines, L1–L3) plants. *ACT2* was used as an internal control. Data are represented as mean ± SEM (n = 3). Statistical differences are indicated with letters ($P < 0.01$, one-way analysis of variance (ANOVA) with Bonferroni post hoc test).
