# Supplementary material for: The Nucleoporin CPR5 Modulates Plant Immunity via Guanylate‐Binding Proteins
Source: Mol Plant Pathol. 2025 Apr 27;26(4):e70086. doi: 10.1111/mpp.70086 (PMC12034427; doi:10.1111/mpp.70086)
Supplement: Supplementary file 3 — Figure S3. Alignment of plant GBPL2, plant RHD3 and animal ATL1 proteins. [file MPP-26-e70086-s006.pdf]

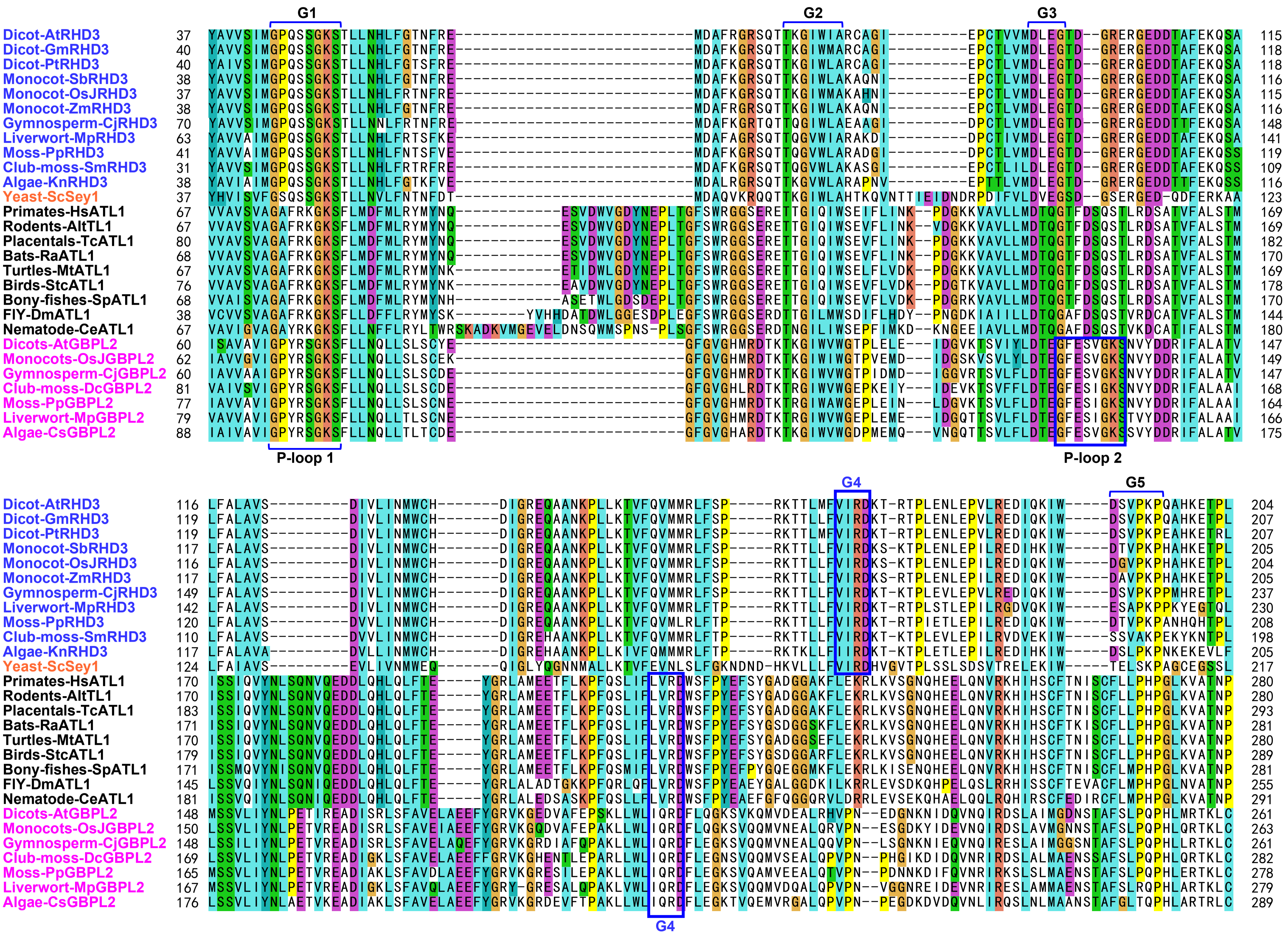

G1
G2
G3
P-loop 1
P-loop 2
G4
G5
Dicot-AtRHD3
Dicot-GmRHD3
Dicot-PtRHD3
Monocot-SbRHD3
Monocot-OsJRHD3
Monocot-ZmRHD3
Gymnosperm-CjRHD3
Liverwort-MpRHD3
Moss-PpRHD3
Club-moss-SmRHD3
Algae-KnRHD3
Yeast-ScSey1
Primates-HsATL1
Rodents-AltTL1
Placentals-TcATL1
Bats-RaATL1
Turtles-MtATL1
Birds-StcATL1
Bony-fishes-SpATL1
FlY-DmATL1
Nematode-CeATL1
Dicots-AtGBPL2
Monocots-OsJGBPL2
Gymnosperm-CjGBPL2
Club-moss-DcGBPL2
Moss-PpGBPL2
Liverwort-MpGBPL2
Algae-CsGBPL2

FIGURE S3. Alignment of plant GBPL2, plant RHD3 and animal ATL1 proteins. Plants include dicots such as *Arabidopsis thaliana* (*At*), *Glycine max* (*Gm*), and *Populus trichocarpa (Pt)*; monocots such as *Oryza sativa Japonica* (*OsJ*), *Sorghum bicolor* (*Sb*), *and Zea mays* (*Zm*); Gymnosperm *Cryptomeria japonica* (*Cj*); club-mosses such as *Diphasiastrum complanatum* (*Dic*) and *Selaginella moellendorffii* (*Sm*); moss *Physcomitrium patens* (*Pp*); liverwort *Marchantia polymorpha* (*Mp*); algae such as *Closterium sp* (*Cs*) and *Klebsormidium nitens* (*Kn*). Yeast includes *Saccharomyces cerevisiae* (*Sc*). Animals include *Caenorhabditis elegans* (*Ce*), *Drosophila melanogaster* (*Dm*), *Homo sapiens* (*Hs*), *Ictidomys tridecemlineatus* (*It*), *Malaclemys terrapin* (*Mt*), *Rousettus aegyptiacus* (*Ra*), *Semicossyphus pulcher* (*Sp*), *Struthio camelus* (*Stc*), and *Tupaia chinensis* (*Tc*). The motifs of G1~G5 boxes and P-loop 1/2 are indicated.
