# Supplementary material for: The Nucleoporin CPR5 Modulates Plant Immunity via Guanylate‐Binding Proteins
Source: Mol Plant Pathol. 2025 Apr 27;26(4):e70086. doi: 10.1111/mpp.70086 (PMC12034427; doi:10.1111/mpp.70086)
Supplement: Supplementary file 4 — Figure S4. The phylogenetic tree of plant GBPL2, plant RHD3 and animal ATL1 proteins. [file MPP-26-e70086-s002.pdf]

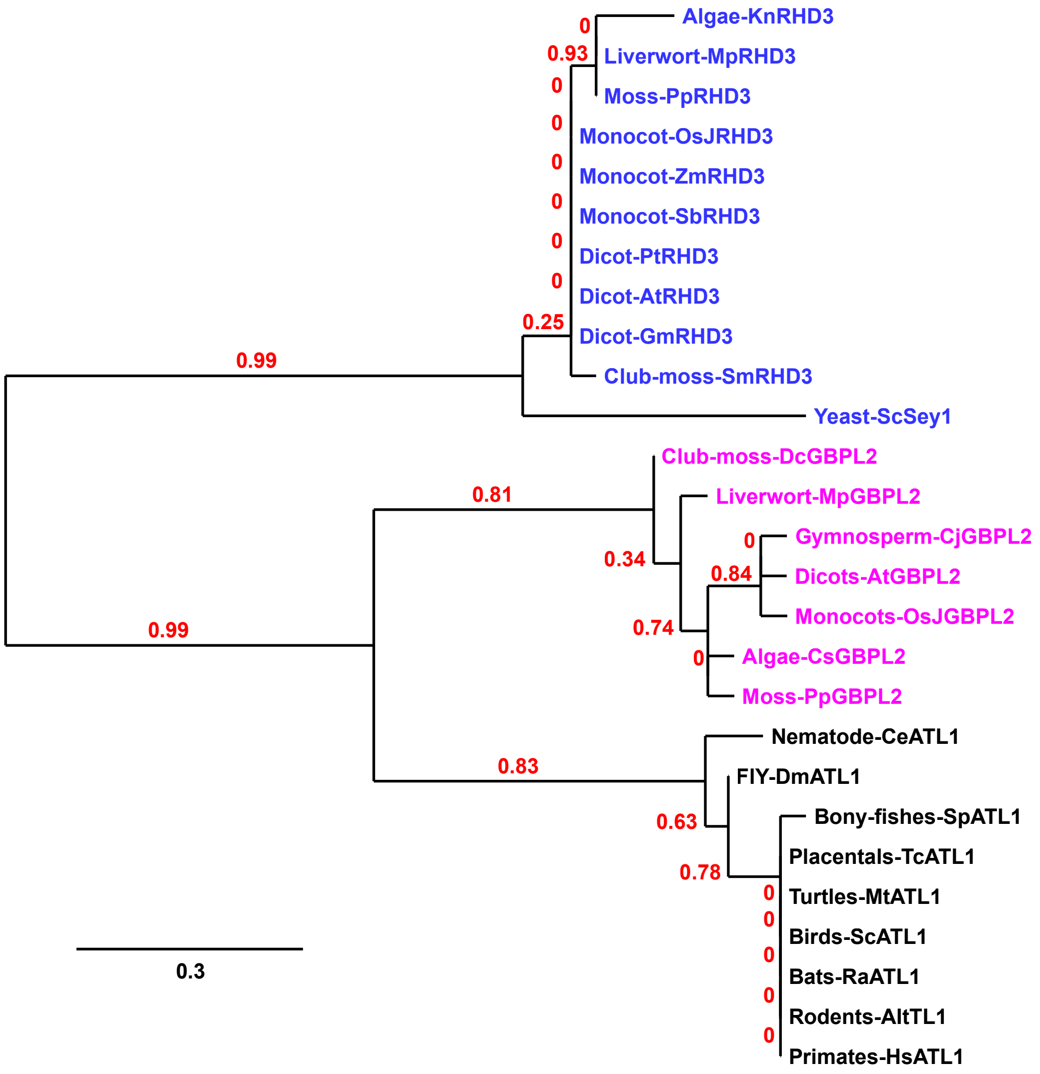

0
Algae-KnRHD3
0.93
Liverwort-MpRHD3
0
Moss-PpRHD3
0
Monocot-OsJRHD3
0
Monocot-ZmRHD3
0
Monocot-SbRHD3
0
Dicot-PtRHD3
0
Dicot-AtRHD3
0
0.25
Dicot-GmRHD3
0.99
Club-moss-SmRHD3
Yeast-ScSey1
Club-moss-DcGBPL2
0.81
Liverwort-MpGBPL2
0
Gymnosperm-CjGBPL2
0.34
0.84
Dicots-AtGBPL2
0.74
Monocots-OsJGBPL2
0.99
0
Algae-CsGBPL2
Moss-PpGBPL2
Nematode-CeATL1
FlY-DmATL1
0.83
Bony-fishes-SpATL1
0.63
Placentals-TcATL1
0.78
0
Turtles-MtATL1
0
Birds-ScATL1
0
Bats-RaATL1
0
Rodents-AltTL1
0
Primates-HsATL1
0.3

FIGURE S4. The phylogenetic tree of plant GBPL2, plant RHD3 and animal ATL1 proteins was constructed at http://www.phylogeny.fr/ based on the sequences of the guanylate-binding protein (GBP) domains in Supplementary Fig. S3.
