# Supplementary material for: The Nucleoporin CPR5 Modulates Plant Immunity via Guanylate‐Binding Proteins
Source: Mol Plant Pathol. 2025 Apr 27;26(4):e70086. doi: 10.1111/mpp.70086 (PMC12034427; doi:10.1111/mpp.70086)
Supplement: Supplementary file 5 — Methods S1. Experimental procedures. [file MPP-26-e70086-s003.pdf]

The nucleoporin CPR5 modulates plant immunity via guanylate-binding proteins

Leiwen Pan[1,†], Shun Peng[1,2,†], Yuehui Zhang[1], Fenghui Xu[1], Xinyi Cai[1], Shen Liang[1], Qi Huang[1], Shilong Yu[1], Shui Wang[1,*]

[1]Shanghai Engineering Research Center of Plant Germplasm Resources, Shanghai Collaborative Innovation Center of Plant Germplasm Resources, College of Life Sciences, Shanghai Normal University, Shanghai 200234, China

[2]National Engineering Laboratory of Crop Stress Resistance, College of Life Science, Anhui Agricultural University, Hefei, Anhui 230036, China

[†]These authors contributed equally: Leiwen Pan and Shun Peng

*Correspondence: shuiwang@shnu.edu.cn

Supporting Information

METHODS S1. Experimental Procedures

Plant materials and growth conditions

All *Arabidopsis thaliana* plants used in this study were in the Columbia (Col-0) background. The condition of growth chamber was set temperature at 22°C and light intensity at 120 μmol m^{-2} s^{-1} generated by Philips Lifemax Cool White fluorescent bulbs with a 16-h light/8-h dark photoperiod. The *cpr5-1* (referred to as *cpr5*) mutant is as described (Wang et al., 2014). The *prl1* mutant is an EMS-mutagenized line (*scpr44*) as described (Peng et al., 2022). Mutants of *fip1* (SALK_099558) and *gbpl2-T* (SALK_121832) were obtained from Arabidopsis Biological Resource Center (ABRC).

Mutant screen and gene cloning

The *cpr5* and *cpr5 scpr23* seeds were mutagenized with ethyl methanesulfonate (EMS). Genetic screens were conducted to identify *suppressor of cpr5* (*scpr*) and *recurrence of cpr5* (*rcpr*) as described (Peng et al., 2022). Next Generation Sequencing (NGS) was utilized to detect single nucleotide polymorphisms (SNPs) responsible for mutations in the *SCPR* genes. The NGS analysis was performed on an Illumina NovaSeq 6000 at Personal Biotechnology Co., Ltd. in Shanghai, China.

The CRISPR/Cas9 editing system

The CRISPR/Cas9 editing system were performed as described (Peng et al., 2022, Yang et al., 2025). All the vectors used for this system were derived from *pCas9-T1-SW* (GenBank accession number: MZ476947).

Trypan blue staining

Trypan blue staining was carried out as described (Peng et al., 2022).

Reverse transcription-quantitative polymerase chain reaction (RT-qPCR)

The RT-qPCR assay were conducted as described (Huang et al., 2024, Zhang et al., 2025). RNA was extracted using TRIzol Reagent (Invitrogen) and cDNA was synthesized using the TransScript One-Step gDNA Removal and cDNA Synthesis SuperMix (TransGen Biotech, Beijing, China). RT-qPCR was performed using the NovoStart SYBR qPCR SuperMix plus (Novoprotein, Shanghai, China) in the CFX Connect Real-Time PCR System (BIO-RAD, Hercules, CA). *ACTIN 2* (*ACT2*, AT3G18780) was used as an internal control. Primers used for qPCR are listed in Table S2.

Pathogen infection

Infection of Arabidopsis plants with *Psm* (*Pseudomonas syringae pv. maculicola* ES4326) and *Psm*/*AvrRpt2* (*Psm* carrying the bacterial effector *AvrRpt2*) was carried out as previously described (Wang et al., 2014). Experiments were conducted 3 times with similar results.

RNA-seq analysis

Two-week-old plants were used for RNA-seq analysis, which was carried out at the Personal Biotechnology Co., Ltd. (Shanghai, China) as described (Peng et al., 2022). Gene expression levels were normalized based on FPKM (fragments per kilobase of exon model per million mapped fragments). DESeq was used for analyzing the significantly differentially expressed genes (DEGs; fold change > 2, $p < 0.05$). Volcano plots illustrating DEGs. Venn diagram proportionally shows the overlap between the altered DEGs of two groups.

Gene ontology (GO) analysis

The gene ontology (GO) analysis was performed using GO enrichment of OmicShare (https://www.omicshare.com/tools/Home/Soft/ gogseasenior).

Confocal laser scanning microscopy (CLSM)

Images of the fluorescent proteins were obtained using a Zeiss LSM 5 PASCAL Confocal Laser Scanning Microscope (Carl Zeiss, Jena, Germany).

Bimolecular fluorescence complementation (BiFC) assays

BiFC assays using luciferase (BiFC-LUC) or yellow fluorescence protein (BiFC-YFP) were carried out as described (Peng et al., 2022).

Phylogenetic tree

The phylogenetic tree of the GBP family proteins was constructed using Phylogeny.fr (http://www.phylogeny.fr/simple_phylogeny.cgi) (Dereeper et al., 2008).

Statistical analysis

Data were analyzed by one-way analysis of variance (ANOVA) with Bonferroni post hoc test or a two-tailed Student's t-test. The experiments were conducted in triplicate. The error bar in the graph represents the standard error of the mean (SEM). For the ANOVA results, the letter above the bar indicates a statistically significant difference between groups at $P < 0.05$. For the Student's t-test results, NS, not significant; *, $P < 0.05$; **, $P < 0.01$; ***, $P < 0.001$; ****, $P < 0.0001$.

Accession numbers

All of RNA-seq data have been deposited in the National Center for Biotechnology Information (NCBI) Sequence Read Archive (SRA) database. RNA-seq data of WT, *cpr5*, *scpr23* and *cpr5 scpr23* plants for differentially expressed genes (DEGs) are available in the SRA database under the Bioproject accession number PRJNA737003.

References

Dereeper, A., Guignon, V., Blanc, G., Audic, S., Buffet, S., Chevenet, F., Dufayard, J. F., Guindon, S., Lefort, V., Lescot, M., Claverie, J. M. & Gascuel, O. (2008) Phylogeny.fr: robust phylogenetic analysis for the non-specialist. *Nucleic Acids Research*, 36, W465-W469.

Huang, S., Zhu, S., Kumar, P. & Macmicking, J. D. (2021) A phase-separated nuclear GBPL circuit controls immunity in plants. *Nature*, 594, 424-429.

Huang, Y., Zhang, Y., Cai, X. & Wang, S. (2025) PURINE PERMEASE 4 regulates plant height in maize. *Journal of Genetics and Genomics*, 52, 446-448

Peng, S., Guo, D., Guo, Y., Zhao, H., Mei, J., Han, Y., Guan, R., Wang, T., Song, T., Sun, K., Liu, Y., Mao, T., Chang, H., Xue, J., Cai, Y., Chen, D. & Wang, S. (2022) CONSTITUTIVE EXPRESSER of PATHOGENESIS-RELATED GENES 5 is an RNA-binding protein controlling plant immunity via an RNA processing complex. *The Plant Cell*, 34, 1724-1744.

Wang, S., Gu, Y., Zebell, S. G., Anderson, L. K., Wang, W., Mohan, R. & Dong, X. (2014) A noncanonical role for the CKI-RB-E2F cell-cycle signaling pathway in plant effector-triggered immunity. *Cell Host & Microbe*, 16, 787-94.

Yang, Y., Chang, H., Pan, L., Guo, D., Peng, S., Mao, T., Zhang, Y. & Wang, S. (2025) Delivery of Marker-Free DNA to Plant Genome by the Transgenic Selection-Associated Fragment Elimination (T-SAFE) System. *Plant Direct*, 9, e70046.

Zhang, Y., Ge, Y., Sun, K., Pan, L., Liang, Z., Wang, P., Cai, Y. & Wang, S. (2025) Identification and application of the heptad repeat domain in the cpr5 protein for enhancing plant immunity. *Molecular Plant Pathology*, 26, e70059.

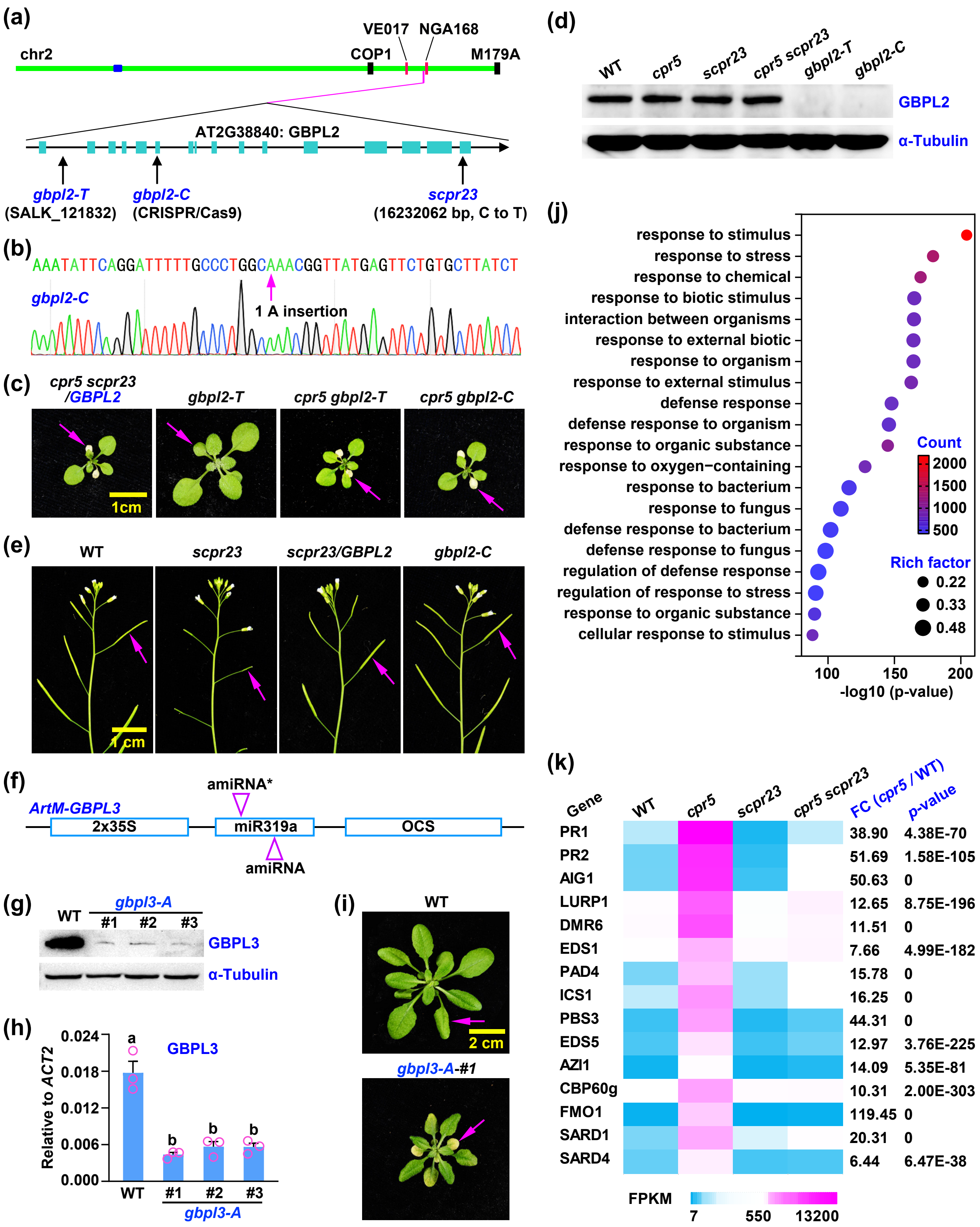
(a)
VE017
NGA168
chr2
COP1
M179A
AT2G38840: GBPL2
gbpl2-T
(SALK_121832)
gbpl2-C
(CRISPR/Cas9)
scpr23
(16232062 bp, C to T)
(b)
AAATATTCAGGATTTTTGCCCTGGCAAACGGTTATGAGTTCTGTGCTTATCT
gbpl2-C
1 A insertion
(c)
cpr5 scpr23 /GBPL2
gbpl2-T
cpr5 gbpl2-T
cpr5 gbpl2-C
1cm
(d)
WT
cpr5
scpr23
cpr5 scpr23
gbpl2-T
gbpl2-C
GBPL2
α-Tubulin
(e)
WT
scpr23
scpr23/GBPL2
gbpl2-C
1 cm
(f)
ArtM-GBPL3
2x35S
amiRNA*
miR319a
amiRNA
OCS
(g)
WT
gbpl3-A
#1
#2
#3
GBPL3
α-Tubulin
(h)
GBPL3
Relative to ACT2
0.024
0.018
0.012
0.006
0.000
a
b
b
b
WT
#1
#2
#3
gbpl3-A
(i)
WT
2 cm
gbpl3-A-#1
(j)
response to stimulus
response to stress
response to chemical
response to biotic stimulus
interaction between organisms
response to external biotic
response to organism
response to external stimulus
defense response
defense response to organism
response to organic substance
response to oxygen−containing
response to bacterium
response to fungus
defense response to bacterium
defense response to fungus
regulation of defense response
regulation of response to stress
response to organic substance
cellular response to stimulus
Count
2000
1500
1000
500
Rich factor
0.22
0.33
0.48
100
150
200
-log10 (p-value)
(k)
Gene
WT
cpr5
scpr23
cpr5 scpr23
FC (cpr5 / WT)
p-value
PR1 38.90 4.38E-70
PR2 51.69 1.58E-105
AIG1 50.63 0
LURP1 12.65 8.75E-196
DMR6 11.51 0
EDS1 7.66 4.99E-182
PAD4 15.78 0
ICS1 16.25 0
PBS3 44.31 0
EDS5 12.97 3.76E-225
AZI1 14.09 5.35E-81
CBP60g 10.31 2.00E-303
FMO1 119.45 0
SARD1 20.31 0
SARD4 6.44 6.47E-38
FPKM
7
550
13200

FIGURE S1. Cloning and characterization of the *SCPR23* gene.

(a) Positional cloning located the *SCPR23* gene within an approximately 846-kb region on chromosome 2, between the cleaved amplified polymorphic sequence (CAPS) marker *VE017* (chr2, 15445461 bp) and the simple sequence length polymorphism (SSLP) marker *NGA168* (chr2, 16291841 bp). Next-generation sequencing (NGS) analysis revealed four single nucleotide polymorphisms (SNPs) in this region. One of these SNPs (chr2, 16232062 bp) resulted in a C to T substitution in the final exon, causing a nonsense mutation (changing the genetic code from CTT to TTT) and leading to an amino acid change from L to F at position 586. This mutation impacts the gene encoding GUANYLATE-BINDING PROTEIN-LIKE 2 (GBPL2). A T-DNA insertion line (SALK_121832), designated as *gbpl2-T*, is located in the first intron of the *GBPL2* gene, while a CRISPR/Cas9-edited line, designated as *gbpl2-C*, is positioned in the sixth exon of this gene.

(b) The CRISPR/Cas9-edited lines of the *GBPL2* gene (*gbpl2-C*).

(c) Two-week-old *cpr5 scpr23*/*GBPL2*, *gbpl2-T*, *cpr5 gbpl2-T*, and *cpr5 gbpl2-C* plants were photographed for early senescence (arrows). *GBPL2*, the *GBPL2* gene; *gbpl2-C*, a CRISPR/Cas9-edited line.

(d) Total proteins of two-week-old WT, *cpr5*, *scpr23*, *cpr5 scpr23*, *gbpl2-T*, and *gbpl2-C* plants were blotted with anti-GBPL2 and anti-α-tubulin. The α-tubulin protein served as a loading control.

(e) The inflorescences of WT, *scpr23*, *scpr23/GBPL2* and *gbpl2-C* were photographed. Siliques are indicated by arrows.

(f) Schematic diagram depicts the structure of *ArtM-GBPL3*, an artificial microRNA construct. The amiRNA and amiRNA* sequences for targeting the *GBPL3* gene were predicted at http://wmd3.weigelworld.org. The primers used for the construction of *ArtM-GBPL3* are listed in S3 Table.

(g) Total proteins of 12-day-old WT and *gbpl3-A* (three lines: #1-#3, as described in Additional file 1: Fig. S2B) plants were blotted with anti-α-Tubulin and anti-GBPL3. The anti-GBPL3 antibody was raised against a synthesized C-terminal fragment of

GBPL3 protein (comprising amino acids 1069-1082: REEERKKQREVTSS) as previously described (Huang et al., 2021). It was produced by Qiwei Yicheng Technology (Beijing, China).

(h) RT-qPCR was carried out on *GBPL3* in 12-day-old WT and *gbpl3-A* (three lines: #1-#3) plants. *ACT2* was used as an internal control. Data are represented as mean ± SEM (n = 3). Statistical differences are indicated with letters ($P < 0.01$, one-way ANOVA with Bonferroni post hoc test).

(i) Twenty-four-day-old WT and *gbpl3-A* plants were photographed for early senescence (arrows).

(j) GO enrichment analysis of 3,542 DEGs which are altered in *cpr5* mutants (*cpr5* versus WT, $P < 0.05$, FC > 2) and depend on *scpr23* (*cpr5 scpr23* versus WT, FC < 2, $P > 0.05$). The bubble chart shows biological process enrichment of DEGs. The y-axis represents biological process. The x-axis represents the enrichment significance (–log10 P-value). Size of the bubble represents rich factor, which is the ratio of the amount of DEGs enriched in a biological process and the amount of all genes annotated in this biological process.

(k) Heatmap showing RNA-seq data of plant immune marker genes, including the defense markers such as *PATHOGENESIS-RELATED 1* (*PR1*), *PR2*, *AVRRPT2-INDUCED GENE 1* (*AIG1*), *LATE UPREGULATED IN RESPONSE TO HYALOPERONOSPORA PARASITICA 1* (*LURP1*), *DOWNY MILDEW RESISTANT 6* (*DMR6*), the core immune regulators such as *ENHANCED DISEASE SUSCEPTIBILITY 1* (*EDS1*) and *PHYTOALEXIN DEFICIENT 4* (*PAD4*), the key SA biosynthesis enzymes and regulators such as *ISOCHORISMATE SYNTHASE 1* (*ICS1*), *AVRPPHB SUSCEPTIBLE 3* (*PBS3*), and EDS5, as well as the key systemic acquired resistance (SAR) regulators such as *AZELAIC ACID INDUCED 1* (*AZI1*), *CALMODULIN BINDING PROTEIN 60-LIKE g* (*CBP60g*), *FLAVIN-DEPENDENT MONOOXYGENASE 1* (*FMO1*), *SAR DEFICIENT 1* (*SARD1*), and *SARD4*. The FC of cpr5/WT as well as its P-values are indicated.

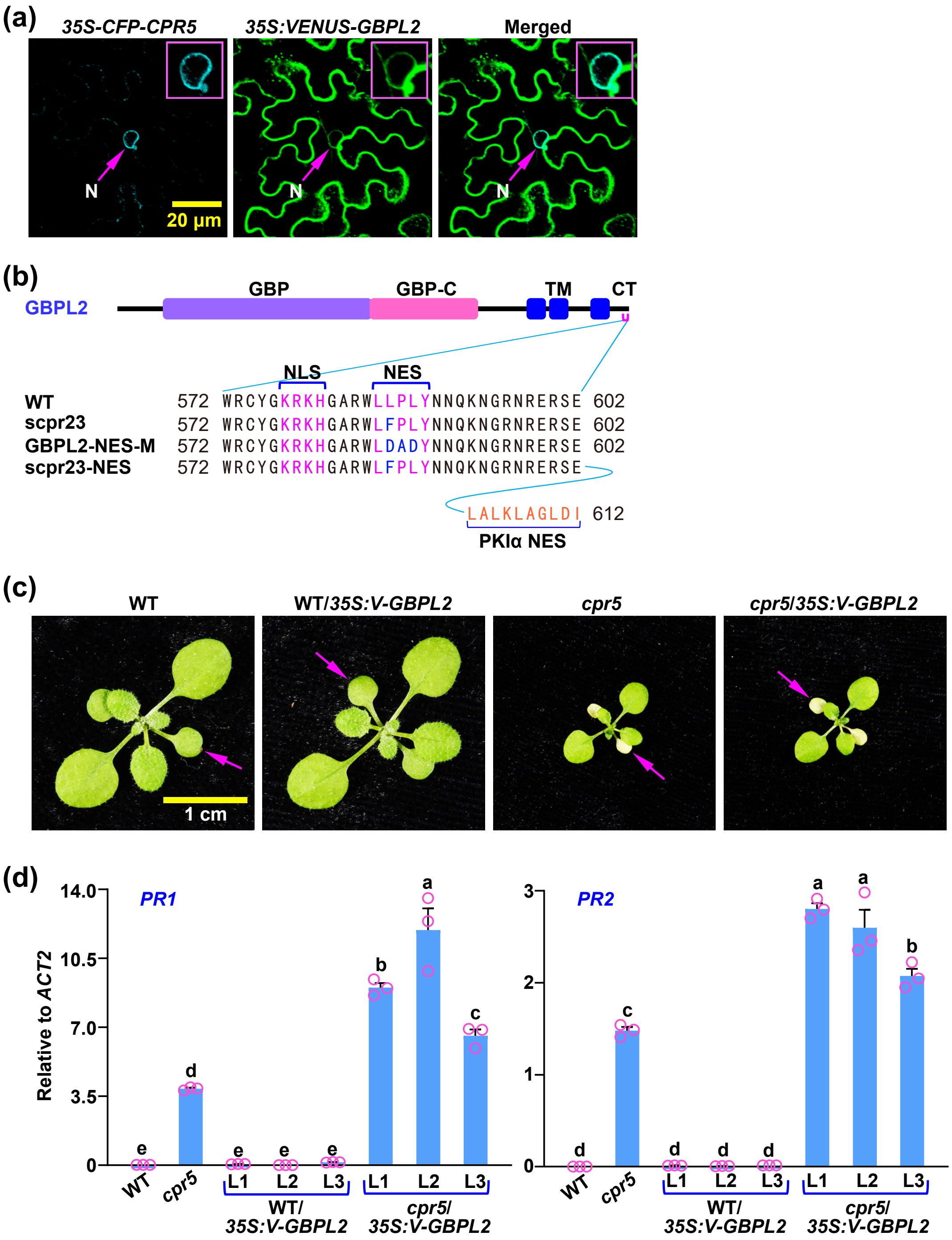

(a)
35S-CFP-CPR5
35S:VENUS-GBPL2
Merged
N
20 µm
(b)
GBPL2
GBP
GBP-C
TM
CT
NLS
NES
WT 572 WRCYGKRKHGARWLLPLYNNQKNGRNRERSE 602
scpr23 572 WRCYGKRKHGARWLFPLYNNQKNGRNRERSE 602
GBPL2-NES-M 572 WRCYGKRKHGARWLDADYNNQKNGRNRERSE 602
scpr23-NES 572 WRCYGKRKHGARWLFPLYNNQKNGRNRERSE
LALKLAGLDI 612
PKIα NES
(c)
WT
WT/35S:V-GBPL2
cpr5
cpr5/35S:V-GBPL2
1 cm
(d)
PR1
PR2
Relative to ACT2
14.0
10.5
7.0
3.5
0
3
2
1
WT
cpr5
L1
L2
L3
WT/ 35S:V-GBPL2
cpr5/ 35S:V-GBPL2

FIGURE S2. Colocalization of CPR5 and GBPL2, and *GBPL2* overexpression in wild-type and *cpr5* plants.

(a) Colocalization analysis was performed by transiently co-expressing *35S:CFP-CPR5* (with CFP fused to the N-terminus of CPR5) and *35S:VENUS-GBPL2* (with VENUS fused to the N-terminus of GBPL2; see in Fig. 2b) in *N. benthamiana* for two days. Inset (pink box): An enlarged view of the nucleus (N, arrow) is shown.

(b) The C-terminus sequences of GBPL2 (WT), scpr23, GBPL2-NES-M (the putative core NES motif "LLPLY" is substituted by "LDADY"), and scpr23-NES (the NES motif, LALKLAGLDI, from human PKIα protein fused to the C-terminus of the scpr23 protein) proteins. The putative NLS and NES motifs are indicated.

(c) Two-week-old wild-type (WT), WT/*35S:V-GBPL2* (expressing the *35S* promoter-driven *VENUS-GBPL2* fusion gene in a WT background), *cpr5*, and *cpr5*/*35S:V-GBPL2* plants were photographed to assess early senescence (arrows).

(d) RT-qPCR analysis of *PR1* (left panel) and *PR2* (right panel) expression in 12-day-old WT, *cpr5*, WT/*35S:V-GBPL2* (three independent transgenic lines, L1–L3), and *cpr5*/*35S:V-GBPL2* (three lines, L1–L3) plants. *ACT2* was used as an internal control. Data are represented as mean ± SEM (n = 3). Statistical differences are indicated with letters ($P < 0.01$, one-way analysis of variance (ANOVA) with Bonferroni post hoc test).

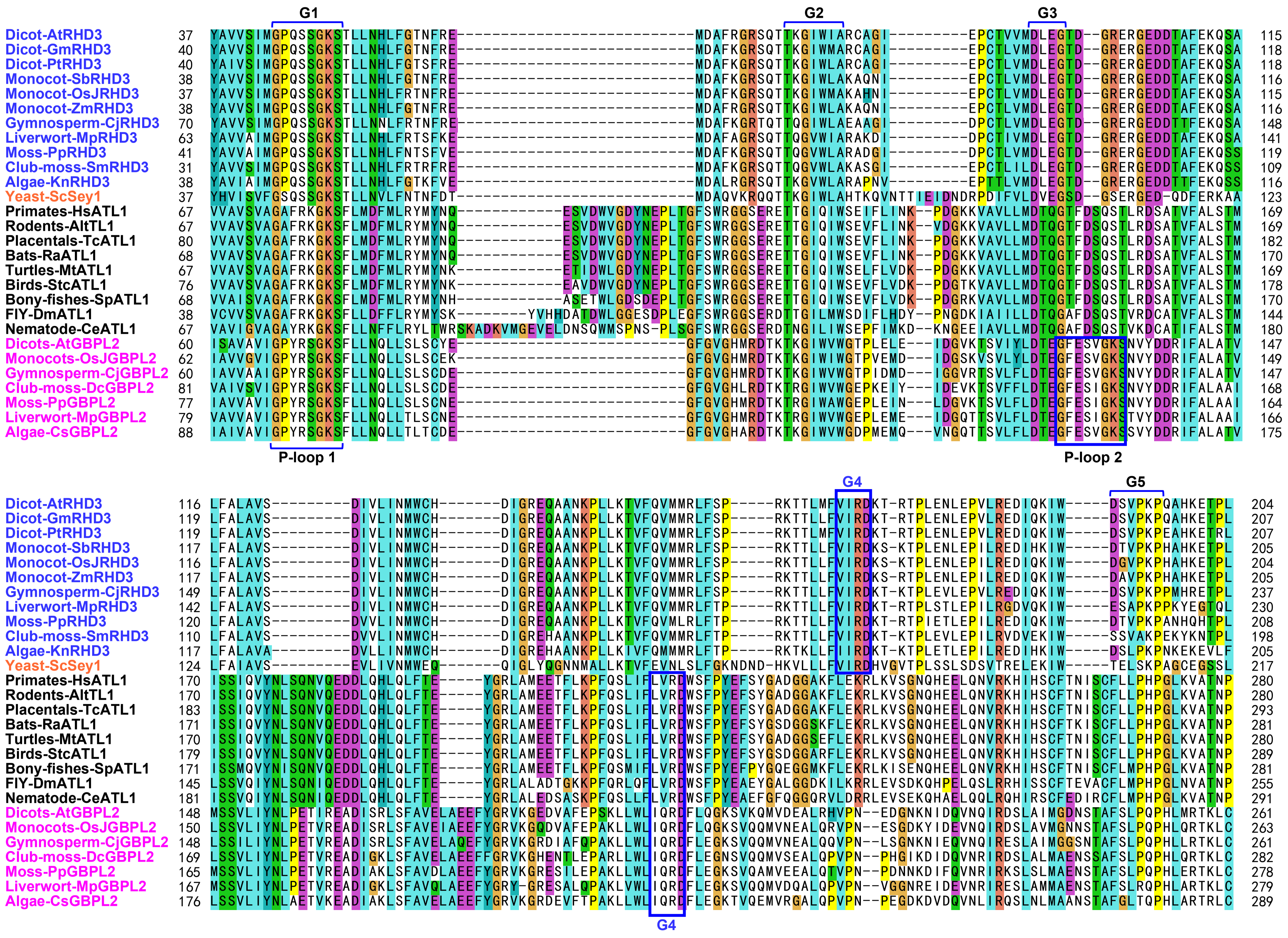

G1
G2
G3
P-loop 1
P-loop 2
G4
G5
Dicot-AtRHD3
Dicot-GmRHD3
Dicot-PtRHD3
Monocot-SbRHD3
Monocot-OsJRHD3
Monocot-ZmRHD3
Gymnosperm-CjRHD3
Liverwort-MpRHD3
Moss-PpRHD3
Club-moss-SmRHD3
Algae-KnRHD3
Yeast-ScSey1
Primates-HsATL1
Rodents-AltTL1
Placentals-TcATL1
Bats-RaATL1
Turtles-MtATL1
Birds-StcATL1
Bony-fishes-SpATL1
FlY-DmATL1
Nematode-CeATL1
Dicots-AtGBPL2
Monocots-OsJGBPL2
Gymnosperm-CjGBPL2
Club-moss-DcGBPL2
Moss-PpGBPL2
Liverwort-MpGBPL2
Algae-CsGBPL2

FIGURE S3. Alignment of plant GBPL2, plant RHD3 and animal ATL1 proteins. Plants include dicots such as *Arabidopsis thaliana* (*At*), *Glycine max* (*Gm*), and *Populus trichocarpa (Pt)*; monocots such as *Oryza sativa Japonica* (*OsJ*), *Sorghum bicolor* (*Sb*), *and Zea mays* (*Zm*); Gymnosperm *Cryptomeria japonica* (*Cj*); club-mosses such as *Diphasiastrum complanatum* (*Dic*) and *Selaginella moellendorffii* (*Sm*); moss *Physcomitrium patens* (*Pp*); liverwort *Marchantia polymorpha* (*Mp*); algae such as *Closterium sp* (*Cs*) and *Klebsormidium nitens* (*Kn*). Yeast includes *Saccharomyces cerevisiae* (*Sc*). Animals include *Caenorhabditis elegans* (*Ce*), *Drosophila melanogaster* (*Dm*), *Homo sapiens* (*Hs*), *Ictidomys tridecemlineatus* (*It*), *Malaclemys terrapin* (*Mt*), *Rousettus aegyptiacus* (*Ra*), *Semicossyphus pulcher* (*Sp*), *Struthio camelus* (*Stc*), and *Tupaia chinensis* (*Tc*). The motifs of G1~G5 boxes and P-loop 1/2 are indicated.

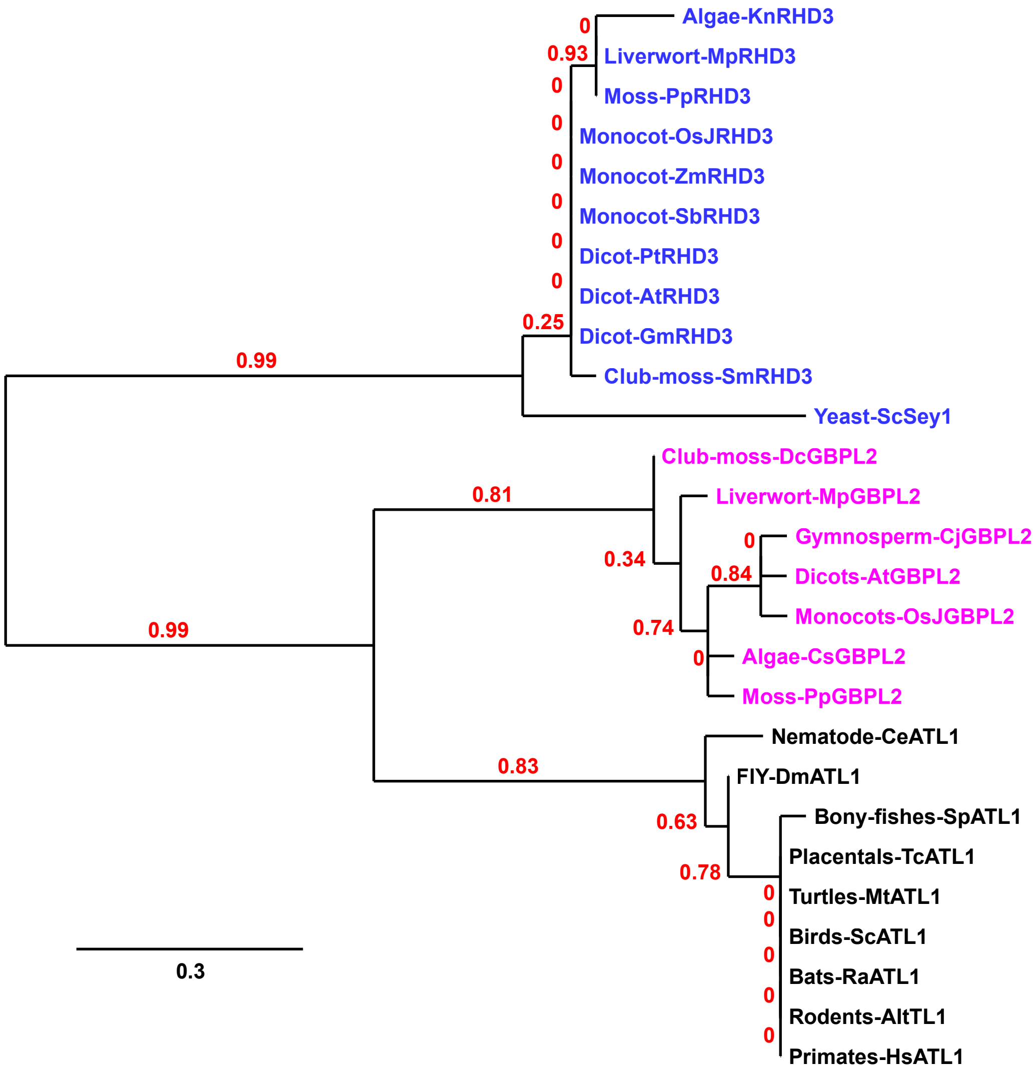

Algae-KnRHD3
Liverwort-MpRHD3
Moss-PpRHD3
Monocot-OsJRHD3
Monocot-ZmRHD3
Monocot-SbRHD3
Dicot-PtRHD3
Dicot-AtRHD3
Dicot-GmRHD3
Club-moss-SmRHD3
Yeast-ScSey1
Club-moss-DcGBPL2
Liverwort-MpGBPL2
Gymnosperm-CjGBPL2
Dicots-AtGBPL2
Monocots-OsJGBPL2
Algae-CsGBPL2
Moss-PpGBPL2
Nematode-CeATL1
FlY-DmATL1
Bony-fishes-SpATL1
Placentals-TcATL1
Turtles-MtATL1
Birds-ScATL1
Bats-RaATL1
Rodents-AltTL1
Primates-HsATL1
0.99
0.93
0.25
0.81
0.34
0.84
0.74
0.83
0.63
0.78
0.3

FIGURE S4. The phylogenetic tree of plant GBPL2, plant RHD3 and animal ATL1 proteins was constructed at http://www.phylogeny.fr/ based on the sequences of the guanylate-binding protein (GBP) domains in Supplementary Fig. S3.

TABLE S1. The exonic and nonsynonymous SNPs around the *SCPR23* gene identified through the next-generation sequencing (NGS) analysis.

**Position**	**Mutation**	**AGI**	**Description**
**Chr2, 15932245 bp**	C→T	AT2G08925	Encoding long noncoding RNA
**Chr2, 16190990 bp**	C→T	AT2G38720	GCT-GTT, A401V, encoding MICROTUBULE-ASSOCIATED PROTEIN 65-5 (MAP65-5)
**Chr2, 16232062 bp**	C→T	AT2G38840	CTT-TTT, L586F, encoding GUANYLATE-BINDING PROTEIN-LIKE 2 (GBPL2)

TABLE S2. The primers used for this study.

Primer name	Sequence (5’-3’)
ACT2-QPCR-F	GGCTCCTCTTAACCCAAAGGC
ACT2-QPCR-R	CACACCATCACCAGAATCCAG
PR1-QPCR-F	CTCATACACTCTGGTGGG
PR1-QPCR-R	TTGGCACATCCGAGTC
PR2-QPCR-F	CAGATTCCGGTACATCAACG
PR2-QPCR-R	AGTGGTGGTGTCAGTGGCTA
