# Supplementary material for: The Nucleoporin CPR5 Modulates Plant Immunity via Guanylate‐Binding Proteins
Source: Mol Plant Pathol. 2025 Apr 27;26(4):e70086. doi: 10.1111/mpp.70086 (PMC12034427; doi:10.1111/mpp.70086)
Supplement: Supplementary file 6 — Table S1. The exonic and nonsynonymous single‐nucleotide polymorphisms (SNPs) around the SCPR23 gene identified through the next‐generation sequencing (NGS) analysis. [file MPP-26-e70086-s005.pdf]

TABLE S1. The exonic and nonsynonymous SNPs around the *SCPR23* gene identified through the next-generation sequencing (NGS) analysis.

Position	Mutation	AGI	Description
**Chr2, 15932245 bp**	C→T	AT2G08925	Encoding long noncoding RNA
**Chr2, 16190990 bp**	C→T	AT2G38720	GCT-GTT, A401V, encoding MICROTUBULE-ASSOCIATED PROTEIN 65-5 (MAP65-5)
**Chr2, 16232062 bp**	C→T	AT2G38840	CTT-TTT, L586F, encoding GUANYLATE-BINDING PROTEIN-LIKE 2 (GBPL2)
